# Injury prevention in fatigue-prone nursing environments: a comprehensive review of strategies centered on work design, human factors engineering, and safety culture

**DOI:** 10.3389/fpubh.2026.1792065

**Published:** 2026-04-13

**Authors:** Lishan Hu, Jinling Ding, Xiao Shen

**Affiliations:** Department of Emergency Medicine, The Second Affiliated Hospital of Zhejiang University School of Medicine, Hangzhou, China

**Keywords:** fatigue risk management, human factors engineering, injury prevention, nurse fatigue, occupational health, patient handling, safety culture, work design

## Abstract

Nurse fatigue is a prevalent and multifactorial occupational health risk that increases the likelihood of work-related injuries and safety incidents, with implications for both workforce well-being and patient care. In fatigue-prone nursing environments, injuries such as slips, sharps injuries, and musculoskeletal disorders related to patient handling represent critical yet often underrecognized consequences of sustained physical and cognitive overload. This review synthesizes findings from occupational health, human factors engineering, sleep medicine, and healthcare quality improvement to examine injury prevention strategies targeting nurse fatigue. Shifting beyond individual-level resilience, the review focuses on system-oriented interventions embedded within work design and care delivery processes. Three interrelated domains are examined: (1) organizational and scheduling strategies, including fatigue risk management systems, shift optimization, protected rest, and acuity-responsive staffing; (2) engineering and ergonomic solutions, such as safe patient handling programs, assistive technologies, environmental optimization, and alarm management; and (3) behavioral and team-based practices, including microbreaks, fatigue-aware communication, training for high-risk tasks, and non-punitive fatigue-related reporting. Across healthcare contexts, implementation is constrained by resource limitations, alarm burden, and deficiencies in safety culture. Evaluation commonly relies on injury incidence, near-miss reporting, sleep quality, and workforce retention, though data standardization remains challenging. Overall, preventing fatigue-related injuries in nursing requires integrated, multidisciplinary, and context-sensitive strategies that align organizational design, engineering controls, and behavioral practices. Embedding fatigue management within healthcare safety culture and policy frameworks is essential to protecting nurses’ health, sustaining workforce stability, and improving patient safety.

## 1 Introduction

Nurse fatigue has become a prominent occupational health concern in modern healthcare systems, with substantial implications for both workforce well-being and patient safety. Fatigue among nurses is a multifactorial phenomenon, shaped by prolonged working hours, high patient-to-nurse ratios, shift work, and the sustained emotional demands of clinical care. Accumulating evidence indicates that chronic fatigue impairs cognitive performance, vigilance, and decision-making, thereby increasing the risk of occupational injuries such as slips, trips, falls, needlestick injuries, and musculoskeletal disorders associated with patient handling (1).

Beyond its immediate safety consequences, nurse fatigue has been consistently associated with reduced job satisfaction, increased burnout, and elevated turnover intentions, all of which threaten workforce stability and healthcare system resilience (2). Fatigue-related cognitive impairment has also been linked to higher rates of clinical errors and adverse events, underscoring its relevance as a patient safety issue rather than solely an individual health concern (3). These challenges were further amplified during the COVID-19 pandemic, which exposed structural vulnerabilities in staffing, scheduling, and occupational health protection and intensified fatigue-related risks among nursing personnel (4).

In response to these growing concerns, the focus of fatigue mitigation has gradually shifted from individual-level coping strategies toward system-oriented approaches that emphasize work design, human factors engineering, and organizational safety culture. Emerging research suggests that interventions targeting team functioning, communication, and resource allocation may attenuate fatigue-related harm more effectively than isolated behavioral strategies (5). Similarly, the integration of ergonomic principles into nursing practice has been shown to reduce physical strain and musculoskeletal injury risk, which are prevalent in fatigue-prone care environments (6).

Against this backdrop, the present review aims to synthesize and integrate strategies for injury prevention in nursing settings characterized by high fatigue exposure, drawing upon a broad range of literature to develop a comprehensive conceptual framework. Drawing on literature from occupational health, human factors engineering, sleep medicine, and healthcare quality improvement, this review examines fatigue as a systemic risk factor embedded within work design and care delivery processes. By integrating insights across organizational, engineering, and behavioral domains, the review seeks to inform the development of comprehensive, implementable interventions that protect nursing staff, enhance patient safety, and support the long-term sustainability of healthcare systems.

**Figure 1:**
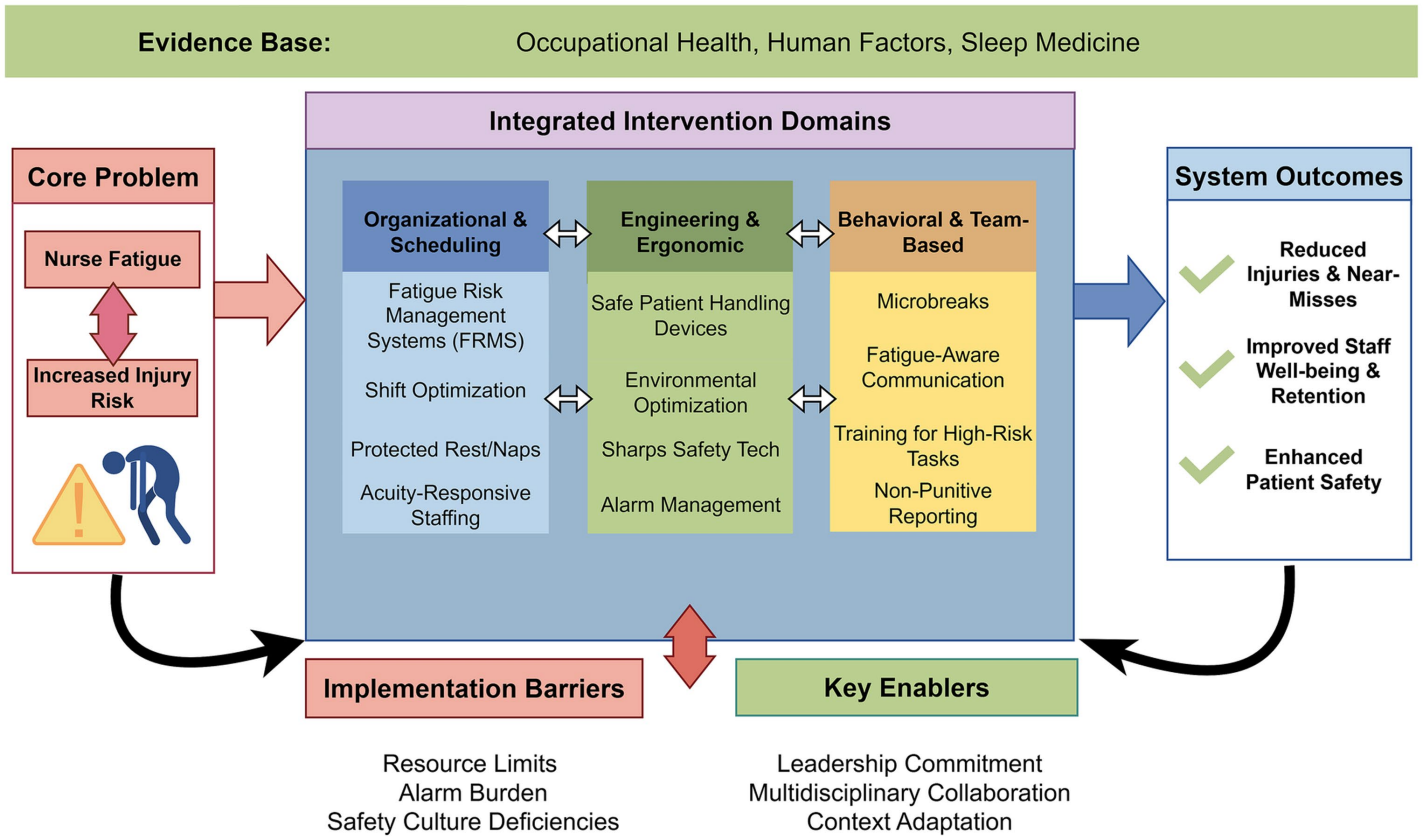
Multilayer strategic model for fatigue-related injury prevention. This conceptual framework illustrates the progression from the core problem (nurse fatigue) through three intervention domains—organizational/scheduling, engineering/ergonomic, and behavioral/team-based—to targeted safety outcomes. The model emphasizes the systemic, integrated nature of effective fatigue mitigation. Figure drawn by Figdraw.com.

To conceptualize this systemic approach, we propose a multilayer strategic model (Figure 1) that integrates organizational, engineering, and behavioral domains to prevent fatigue-related injuries, illustrating the progression from the core problem to targeted interventions and desired outcomes. Before proceeding, brief definitional clarification of key terms is warranted to ensure consistency throughout this review. Fatigue refers to a state of physical and/or mental exhaustion resulting from prolonged work, insufficient recovery, or circadian disruption, which impairs performance and increases injury risk. This is distinct from sleepiness, which specifically reflects the propensity to fall asleep. Occupational fatigue encompasses both acute (shift-level) and chronic (cumulative) dimensions, whereas burnout—while related—is a psychological syndrome characterized by emotional exhaustion, depersonalization, and reduced personal accomplishment, typically resulting from chronic workplace stress rather than sleep or recovery insufficiency alone. Regarding intervention terminology, fatigue mitigation, fatigue management, and fatigue risk management are used interchangeably to refer to strategies aimed at reducing fatigue occurrence or its consequences, while injury prevention specifically denotes the outcome of interest. Where distinctions among these terms are conceptually important, they are noted in the text.

## 2 Methods

### 2.1 Review design

This is a narrative review (also referred to as a traditional or critical review) designed to synthesize evidence from a broad range of disciplines—including occupational health, human factors engineering, sleep medicine, and healthcare quality improvement. Unlike systematic reviews that aim to answer a narrow clinical question through exhaustive meta-analysis, this review adopts a thematic and conceptual approach. Its primary goal is to integrate diverse streams of literature to propose a comprehensive, three-domain conceptual framework (organizational, engineering, and behavioral) for understanding and preventing fatigue-related injuries in nursing. This approach is particularly suited to topics that are multifaceted and where evidence comes from heterogeneous methodologies that are not amenable to quantitative pooling. The synthesized findings are presented in Tables 1–5, each of which integrates evidence from this narrative review across the three domains.

**Table 1:** Core intervention strategies for preventing fatigue-related injuries in nursing.

Domain	Category	Specific intervention/Approach	Primary mechanism of action	Outcome type	Representative outcomes/evidence highlights	Key references
Organizational & scheduling strategies	Fatigue Risk Management System (FRMS)	Data-informed risk assessment; biomathematical modeling; fatigue self-reporting	Enables proactive, system-level identification and mitigation of fatigue risks	Occupational injury reduction; Fatigue/sleep metrics	Reduces fatigue-related errors; effectiveness contingent on robust safety culture and leadership commitment	(7, 9, 12, 13)
	Shift Pattern Optimization	Limiting consecutive night shifts; avoiding quick returns (<11 h); participatory scheduling	Minimizes circadian disruption and cumulative sleep debt	Occupational injury reduction; Fatigue/sleep metrics; Patient safety	Improves sleep quality and alertness; associated with reduced medication and sharps injury rates	(14, 15, 17, 21, 22)
	Protected Rest & Strategic Napping	Institutional policies endorsing short naps (10–30 min); designated rest areas	Prevents sustained workload overload and fatigue accumulation	Fatigue/sleep metrics; Patient safety	Enhances vigilance, motor function, and decision-making; effectiveness depends on cultural acceptance	(24–26)
	Acuity-Responsive Staffing	Validated tools to match nurse staffing with real-time patient care needs	Prevents sustained workload overload and fatigue accumulation	Patient safety; Occupational injury reduction	Associated with reduced adverse events (e.g., inpatient falls, infections, missed care)	(27–32)
Engineering & ergonomic solutions	Safe Patient Handling & Assistive Devices	Ceiling lifts; air-assisted transfer systems; back-support exoskeletons	Reduces biomechanical load during patient handling tasks	Occupational injury reduction (MSDs)	Decreases spinal loading and muscle activation; lowers musculoskeletal disorder risk; underutilized due to workflow barriers	(40–44)
	Environmental Optimization	Ergonomic unit layout; slip-resistant flooring; optimized lighting	Reduces unnecessary movement, postural strain, and environmental hazards	Occupational injury reduction (slips/falls); Fatigue/sleep metrics	Improves workflow efficiency; mitigates slip/trip risks and visual fatigue	(45–49)
	Sharps Injury Prevention	Safety-engineered devices (SEDs); accessible and well-maintained disposal systems	Creates passive safety barriers and supports safe work practices	Occupational injury reduction (sharps)	Reduces injury incidence; effectiveness limited by incomplete adoption and inconsistent training	(50–57)
	Alarm & Cognitive Load Management	Intelligent alarm filtering; clinically relevant prioritization algorithms	Reduces non-actionable alerts and mitigates cognitive overload	Fatigue/sleep metrics; Patient safety	Decreases alarm fatigue; supports sustained attention and clinical decision-making	(66–71, 111)
Behavioral & team practices	Microbreaks & Self-Regulation	Brief, self-initiated rest periods with stretching or posture changes	Intermittently replenishes attentional and physical resources	Fatigue/sleep metrics; Occupational injury reduction	Reduces musculoskeletal discomfort and perceived fatigue; requires supportive safety climate	(72–77)
	Fatigue-Aware Communication	Open disclosure of fatigue status during handovers and team huddles	Enhances shared situational awareness and adaptive task allocation	Patient safety; Implementation outcomes	Facilitates team-based risk mitigation; strengthens psychological safety	(78–81, 84)
	Fatigue-Informed Training	Simulation-based training incorporating fatigue awareness and human factors	Enhances skill retention and adaptive decision-making under fatigue conditions	Occupational injury reduction; Patient safety	Improves compliance with safety protocols during high-risk tasks	(86–90)
	Non-Punitive Reporting Systems	Blame-free reporting of fatigue-related incidents and near-misses	Enables organizational learning from systemic vulnerabilities	Implementation outcomes; Learning	Identifies patterns in scheduling, staffing, and environmental contributors to fatigue	(91–93, 114)

This table synthesizes findings from a narrative review of the literature, integrating evidence across organizational, engineering, and behavioral domains. Outcome types are categorized as: occupational injury reduction (specific injury types indicated in parentheses), patient safety, fatigue/sleep metrics, and implementation outcomes. This table summarizes intervention strategies for preventing fatigue-related injuries in nursing, organized within three interrelated domains: organizational/scheduling, engineering/ergonomic, and behavioral/team practices. For each strategy, the primary mechanism of action is described, along with representative outcomes and supporting references. An additional column specifies the outcome type—occupational injury, patient safety, fatigue/sleep metrics, or implementation outcomes—to enhance clarity regarding the intended effects of each intervention.

**Table 2:** Implementation barriers to fatigue-related injury prevention strategies.

Barrier category	Specific barrier	Description	Affected domain(s)	Potential mitigation strategies	Key references
Resource constraints	Financial limitations	Insufficient budget for equipment, technology, and program infrastructure	Organizational, Engineering	Prioritize low-cost, high-impact interventions (e.g., scheduling optimization, in-situ simulation); phased implementation	(105–110)
	Staffing shortages	Inadequate nurse-to-patient ratios; limited replacement capacity for rest breaks	Organizational	Integrate workforce planning with fatigue risk assessment; flexible scheduling models	(105, 110)
	Training capacity limitations	Insufficient time and resources for comprehensive staff education	All domains	Leverage digital training platforms; embed fatigue education into existing curricula	(106, 107)
	Infrastructure deficits	Lack of designated rest spaces; inadequate device availability	Organizational, Engineering	Incremental infrastructure investment; creative use of existing space	(105)
Workload & system factors	Alarm fatigue	Excessive non-actionable alarms leading to desensitization and delayed responses	Engineering, Behavioral	Implement intelligent alarm filtering; standardize alarm parameters; clinician education	(66, 67, 69, 111)
	Information overload	Fragmented device ecosystems; excessive documentation requirements	All domains	Integrate systems; streamline workflows; human-centered technology design	(69, 111–113)
	Physical workload intensity	High patient handling demands; prolonged standing	Engineering, Behavioral	Safe patient handling programs; microbreak promotion; ergonomic redesign	(40–44, 72–77)
Safety culture deficiencies	Punitive norms	Blame-oriented responses to errors and near-misses	All domains	Leadership development; just culture training; transparent reporting systems	(114–117)
	Stigma surrounding fatigue	Fatigue framed as individual weakness rather than occupational hazard	All domains	Normalize fatigue discussions; leadership modeling; education campaigns	(114, 118)
	Weak leadership engagement	Limited visibility and commitment from unit and organizational leaders	All domains	Leadership accountability metrics; safety rounds; visible endorsement of fatigue policies	(115, 116, 119)
	Hierarchical barriers	Communication hierarchies that discourage frontline voice	Behavioral	Structured communication tools; psychological safety training; multidisciplinary briefings	(114, 117)
Technology acceptance barriers	Workflow disruption concerns	Perceived interference with clinical tasks and patient care	Engineering, Behavioral	User-centered design; frontline involvement in technology selection	(44, 63)
	Inadequate training	Insufficient education on proper device use and benefits	Engineering, Behavioral	Competency-based training; peer champions; refresher sessions	(44, 50, 51)
	Device discomfort or impracticality	Poor fit, weight, or usability of PPE and assistive devices	Engineering	Ergonomic design specifications; staff input in procurement decisions	(58–63)
Contextual adaptation needs	Resource heterogeneity	Variation in availability of financial, technological, and human resources across settings	All domains	Flexible implementation frameworks; tiered intervention packages	(124–128)
	Regulatory variability	Differences in working time regulations and occupational health standards	Organizational	Context-sensitive policy interpretation; alignment with local requirements	(124, 125)
	Cultural norms	Variation in attitudes toward shift work, fatigue, and help-seeking	All domains	Culturally tailored education; local champions; adaptation of communication strategies	(126–128)

This table synthesizes major barriers that impede the implementation of fatigue mitigation strategies in nursing environments. Barriers are organized by category—resource constraints, workload and system factors, safety culture deficiencies, technology acceptance barriers, and contextual adaptation needs. For each barrier, the affected domain(s) within the three-domain framework are identified, and potential mitigation strategies are proposed based on the reviewed literature.

**Table 3:** Evaluation challenges in fatigue-related injury prevention research and practice.

Challenge category	Specific challenge	Description	Impact on evaluation	Potential mitigation strategies	Key references
Reporting & measurement bias	Underreporting of incidents	Fear of blame, reporting fatigue, unclear reporting pathways	Deflates injury and near-miss rates; obscures true safety status	Non-punitive reporting culture; simplified reporting interfaces; anonymous reporting options	(54–56, 114, 120)
	Social desirability bias	Reluctance to admit fatigue or errors due to professional norms	Inflates self-reported alertness; underestimates fatigue prevalence	Anonymous surveys; validated instruments; normalization of fatigue as occupational reality	(120, 121)
	Recall bias	Retrospective self-report of fatigue, sleep, or events	Reduces accuracy of exposure-outcome associations	Real-time data capture; ecological momentary assessment; objective measures where feasible	(121, 122)
Definitional inconsistency	Variable fatigue operationalization	Diverse instruments and cut-points for fatigue measurement	Limits cross-study comparability; impedes meta-analysis	Adoption of validated core measures; consensus on minimum data elements	(See Table 1 notes, (122))
	Heterogeneous injury definitions	Differences in what constitutes a “reportable” injury across settings and studies	Complicates benchmarking and synthesis	Alignment with regulatory standards (e.g., OSHA); clear specification in study protocols	(120, 123)
	Conflated outcomes	Mixing occupational injuries, patient safety events, and process measures	Obscures mechanism-specific intervention effects	Explicit outcome classification (as in Table 1); stratified reporting	(120)
Methodological limitations	Predominance of observational designs	Limited randomized controlled trials due to ethical and practical constraints	Restricts causal inference about intervention effectiveness	Quasi-experimental designs; interrupted time series; rigorous confounding control	(121, 123)
	Short follow-up periods	Evaluation limited to immediate post-implementation	Misses long-term sustainability and delayed effects	Extended follow-up; periodic reassessment; sustainability metrics	(122, 123)
	Single-site studies	Limited generalizability to diverse contexts	Reduces external validity and implementation guidance	Multi-site collaborations; implementation science frameworks; contextual factor documentation	(124–128)
Data infrastructure limitations	Fragmented reporting systems	Disconnected occupational health, patient safety, and human resources data	Hinders integrated analysis of fatigue-safety relationships	Integrated data platforms; common identifiers; interoperable systems	(120, 122)
	Paper-based or legacy systems	Outdated data collection methods	Increases reporting burden; delays data availability	Electronic incident reporting; mobile-friendly interfaces; automated data capture	(120, 123)
	Limited technology validation	Insufficient evidence on accuracy of wearable and digital fatigue measures	Constrains confidence in real-time monitoring applications	Rigorous validation studies; comparison with gold standards; contextual performance assessment	(64, 65, 134–136)

Barriers and challenges identified through a narrative synthesis of the literature, reflecting themes across diverse study designs and healthcare contexts. Affected domains refer to the three-domain framework (organizational/scheduling, engineering/ergonomic, behavioral/team practices) used throughout this review. This table summarizes key challenges encountered in evaluating fatigue-related injury prevention interventions. Challenges are organized into four categories: reporting and measurement bias, definitional inconsistency, methodological limitations, and data infrastructure limitations. For each challenge, the impact on evaluation validity is described, along with potential mitigation strategies to improve the quality and interpretability of future research and practice-based evaluation.

**Table 4:** Future research and development priorities.

Priority area	Critical Research questions/Development goals	Anticipated contribution to practice or policy	Key references
Multidisciplinary collaboration models	How to structure and sustain effective teams integrating occupational health, ergonomics, sleep science, and healthcare management?	Facilitates translation of findings into context-sensitive, system-level interventions; enhances coordinated risk management	(129–133)
Emerging technology validation	What is the clinical validity, usability, and safety impact of wearable sensors, AI-driven fatigue prediction, and robotics in real-world nursing environments?	Enables real-time fatigue monitoring and proactive workload management; reduces physical and cognitive burden	(64, 65, 134–136)
Personalized fatigue management	How to integrate individual factors (chronotype, health status, recovery capacity) into FRMS without shifting undue responsibility onto individuals?	Develops equitable, tailored strategies that enhance both safety and staff well-being within system-level frameworks	(7, 137–141)
Safety culture & behavior change	What are the mechanisms through which leadership behaviors, psychological safety, and organizational norms influence fatigue-related practices?	Informs targeted interventions to sustain proactive safety behaviors and a non-punitive reporting culture	(142–149)
Economic evaluation & policy support	What is the long-term cost-effectiveness of integrated fatigue mitigation strategies? How can regulatory frameworks best incentivize and sustain implementation?	Provides evidence for sustainable investment; supports development of policies that institutionalize fatigue risk management	(150–153)

Priority areas identified from a narrative review of current evidence gaps and emerging trends in fatigue-related injury prevention. Research questions are framed to address both knowledge generation and translation into practice. This table identifies priority areas for future research and development to advance the science and practice of fatigue-related injury prevention. For each priority area, critical research questions or development goals are articulated, along with the anticipated contribution to practice or policy. Priorities encompass multidisciplinary collaboration, emerging technology validation, personalized fatigue management, safety culture and behavior change, and economic evaluation with policy support.

**Table 5:** Integrated recommendations for practice and policy.

Target level	Core action items	Rationale and expected impact	Supporting evidence (Domain)
Organizational leadership	1. Implement a data-driven Fatigue Risk Management System (FRMS) with continuous monitoring 2. Adopt evidence-based shift scheduling policies (e.g., limit quick returns and consecutive night shifts) 3. Establish and resource protected rest periods with designated nap spaces	Establishes foundational, system-level infrastructure for proactive fatigue risk identification and mitigation; moves beyond individual-level coping	Organizational & Scheduling Strategies
Engineering & work design	1. Invest in and normalize use of assistive patient handling technologies 2. Optimize unit layout and environmental features (lighting, flooring) to reduce physical hazards 3. Implement intelligent alarm management systems to reduce cognitive load	Reduces physical and cognitive demands of the work environment; creates passive safety barriers that function independently of individual vigilance	Engineering & Ergonomic Solutions
Team & behavioral support	1. Foster a culture of open, non-punitive fatigue communication and incident reporting 2. Integrate microbreaks and fatigue awareness into routine team huddles and training curricula 3. Provide accessible psychological support and sleep health resources	Empowers frontline teams to self-regulate and adapt; leverages collective vigilance, peer support, and shared accountability	Behavioral & Team Practices
Engineering & work design	1. Align nurse staffing levels dynamically with real-time patient acuity 2. Address safety culture deficiencies through leadership development and transparent feedback mechanisms 3. Adapt implementation strategies to local resource availability, regulatory context, and cultural norms 4. Establish evaluation frameworks with standardized outcome metrics (see Section 6.6)	Ensures interventions are feasible, sustainable, and contextually appropriate; bridges the gap between evidence and real-world practice; enables continuous quality improvement	Cross-cutting (Staffing, Culture, Implementation, Evaluation)

Recommendations synthesized from a narrative review of evidence across organizational, engineering, and behavioral domains, tailored to diverse implementation contexts. Implementation success depends on simultaneous attention to technical, cultural, and contextual factors. This table provides actionable recommendations derived from the synthesized literature, structured across four target levels: organizational leadership, engineering and work design, team and behavioral support, and system-enabling conditions. Each recommendation is accompanied by its rationale and expected impact, as well as the primary evidence domain from which it is drawn. The recommendations are intended to guide the development of comprehensive, context-sensitive fatigue mitigation policies and programs.

### 2.2 Search strategy

A comprehensive literature search was conducted in the following databases: PubMed (via PubMed interface), CINAHL (via EBSCOhost), PsycINFO (via EBSCOhost), and Web of Science (via Clarivate platform). The search was initially executed on October 15, 2025, and updated on December 20, 2025, to capture recently published literature. The search was limited to peer-reviewed articles published in English, with no lower date limit applied.

The search strategy combined controlled vocabulary (where applicable) and free-text terms related to four conceptual blocks: nursing population, fatigue, injury prevention, and intervention domains. The full search strings as executed in each database are reported below, See Supplementary Material 1. In addition to database searches, the reference lists of all included studies and relevant review articles were hand-searched to identify additional eligible publications. The search was restricted to English-language publications due to resource constraints for translation.

### 2.3 Study selection

The selection of literature was guided by the review’s conceptual framework. Given the narrative nature of this synthesis, no formal quality assessment tools were applied to exclude studies. Instead, emphasis was placed on thematic relevance, conceptual contribution, and methodological diversity. Studies were prioritized if they: (a) provided empirical data on nursing populations, (b) evaluated interventions or risk factors related to the three proposed domains, or (c) offered theoretical or mechanistic insights into fatigue and injury. This inclusive approach allows for the integration of quantitative, qualitative, and simulation-based evidence, providing a holistic understanding of the field.

**Figure 2:**
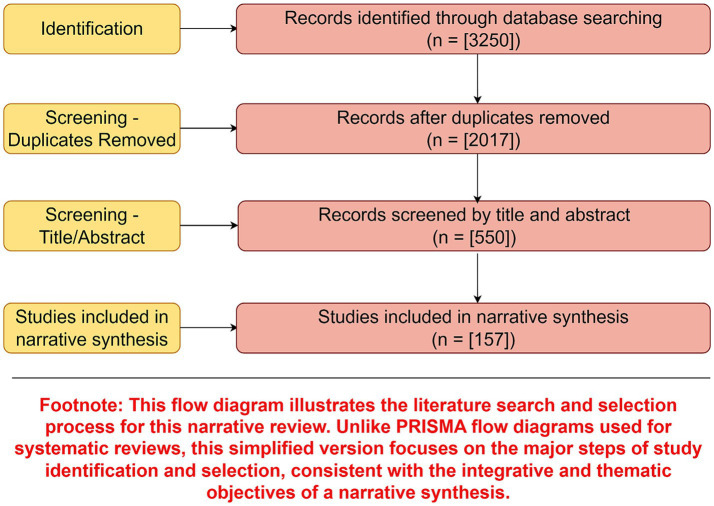
Literature search and selection process. Flow diagram summarizing the identification, screening, and inclusion of studies for this narrative review. A total of 3,250 records were initially identified, with 157 studies included in the final synthesis informing Tables 1–5. Given the narrative methodology, exclusion reasons at full-text stage are not quantitatively reported. Figure drawn by Figdraw.com. This flow diagram illustrates the literature search and selection process for this narrative review. Unlike PRISMA flow diagrams used for systematic reviews, this simplified version focuses on the major steps of study identification and selection, consistent with the integrative and thematic objectives of a narrative synthesis.

The literature search and selection process is summarized in Figure 2. A total of 3,250 records were initially identified through database searching. After removing duplicates and screening by title and abstract, 157 studies were included in the narrative synthesis. This process reflects the breadth of literature informing the organizational, engineering, and behavioral domains of the review.

### 2.4 Screening process

The screening process was conducted in three stages: duplicate removal, title/abstract screening, and full-text review. Duplicates were identified and removed using EndNote X9 reference management software, first through automated duplicate detection and subsequently through manual verification.

Title and abstract screening was performed independently by two reviewers (LH and JD). Each reviewer evaluated records against preliminary eligibility criteria based on the review’s conceptual framework. Records were retained if they: (a) involved nursing populations or healthcare workers with separate nurse-specific analyses; (b) addressed fatigue, sleep, or shift work in relation to occupational injury prevention; or (c) described interventions or risk factors within the organizational, engineering, or behavioral domains. Disagreements between reviewers were resolved through discussion or, when consensus could not be reached, by consultation with a third reviewer (XS).

Full-text articles were retrieved for all records passing title/abstract screening. Two reviewers independently assessed full texts against the final eligibility criteria (detailed in Section 2.5). Reasons for exclusion at the full-text stage were documented, although formal exclusion counts are not reported given the narrative, concept-building nature of this review. The final set of included studies comprised 157 articles that substantively informed the development of the three-domain conceptual framework and the synthesis presented in Tables 1–5.

### 2.5 Eligibility criteria

Consistent with the narrative and conceptual orientation of this review, eligibility criteria were designed to be inclusive while maintaining thematic focus. Studies were considered eligible if they met the following criteria:

Population: Studies primarily involving registered nurses, licensed practical nurses, nursing assistants, or student nurses in clinical practice settings were included. Studies involving mixed healthcare worker populations (e.g., physicians, nurses, allied health professionals) were included if findings specific to nursing personnel could be extracted separately or if nurses constituted the majority of the sample.

Setting: Eligible care settings included acute care hospitals (e.g., intensive care units, emergency departments, medical-surgical units), long-term care facilities (e.g., nursing homes, skilled nursing facilities), and community-based nursing settings (e.g., home healthcare, outpatient clinics). Non-clinical settings (e.g., academic institutions without clinical practice components) were excluded.

Outcomes: Studies were included if they addressed one or more of the following outcome categories: (1) occupational injuries to nurses (e.g., needlestick/sharps injuries, slips/trips/falls, musculoskeletal disorders, patient handling injuries); (2) patient safety incidents potentially linked to nurse fatigue (e.g., medication errors, falls, healthcare-associated infections); (3) fatigue or sleep-related measures (e.g., subjective fatigue, sleep quality, sleep duration, alertness); or (4) implementation outcomes relevant to fatigue interventions (e.g., feasibility, acceptability, barriers).

Study designs: All primary study designs were eligible, including randomized controlled trials, quasi-experimental studies, cohort studies, cross-sectional studies, qualitative studies, and simulation-based studies. Systematic reviews and meta-analyses were used as source material for identifying primary studies but were not themselves included in the synthesis to avoid duplication. Theoretical papers, expert consensus statements, and narrative reviews were included when they provided conceptual or mechanistic insights not available in empirical studies.

Intervention focus: For intervention-focused recommendations, priority was given to studies evaluating specific interventions within the three domains (organizational/scheduling, engineering/ergonomics, behavioral/team practices). Observational studies examining risk factor–outcome associations were used to inform the rationale for interventions and to identify implementation considerations, rather than to infer intervention effectiveness directly.

No formal quality appraisal was conducted, consistent with the narrative review methodology, as the goal was conceptual integration rather than evidence grading. However, study limitations noted in source publications were considered during interpretation and are reflected in the discussion.

## 3 Organizational and scheduling strategies in care environments prone to fatigue

**Figure 3:**
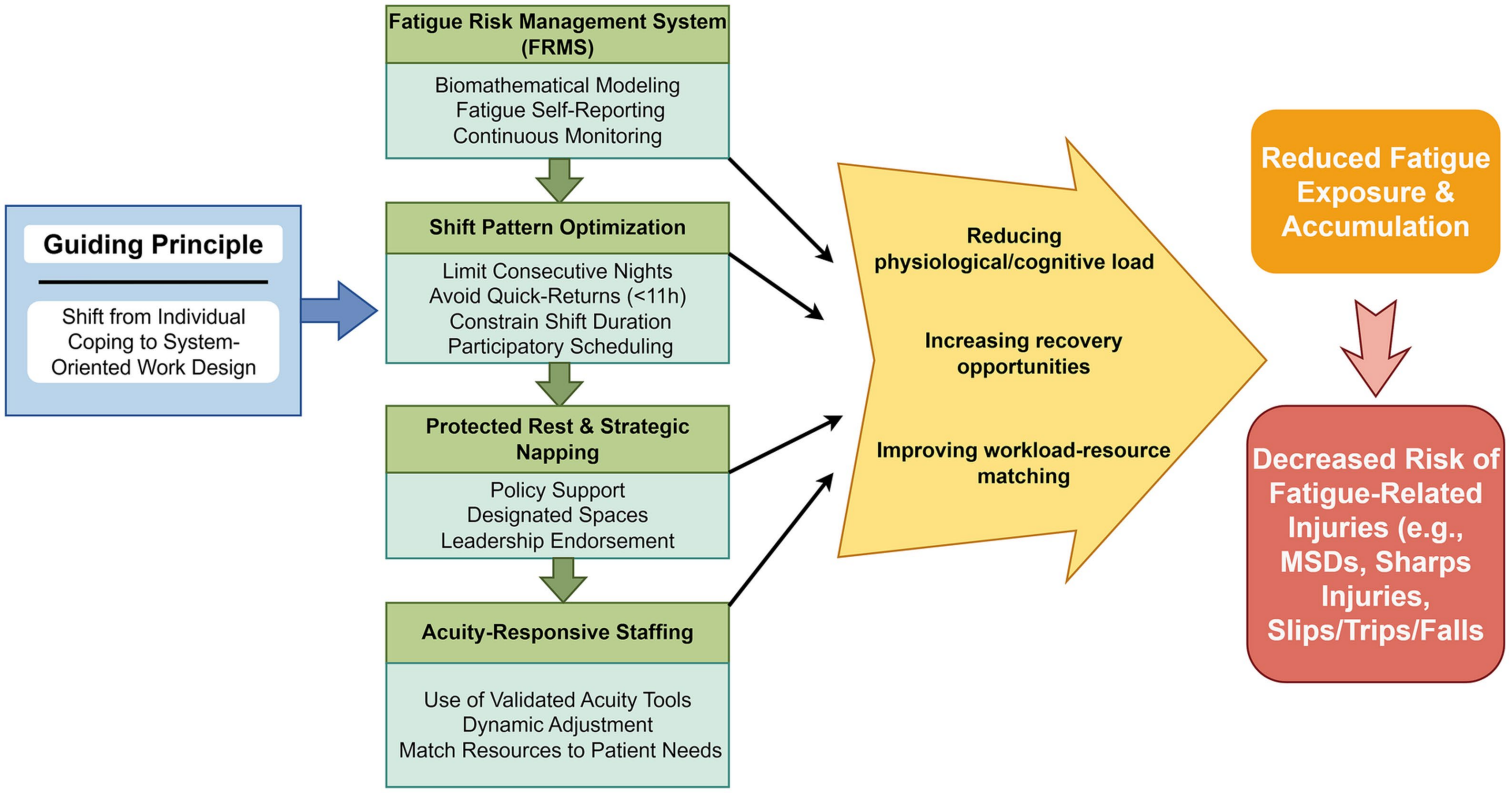
Organizational strategies pathway to fatigue and injury reduction. This logic model depicts the causal pathway from system-oriented design principles, through specific scheduling and staffing interventions, to their cumulative impact on fatigue accumulation and injury risk. Figure drawn by Figdraw.com.

The implementation of evidence-based organizational strategies follows a coherent pathway to reduce fatigue and injury risk. Figure 3 delineates this logical flow, from the foundational principle of system-oriented design, through specific scheduling and staffing levers, to their cumulative impact on fatigue and injury prevention.

### 3.1 Construction and application of fatigue risk management systems (FRMS)

Fatigue Risk Management Systems (FRMS) represent a structured, data-informed approach to identifying, assessing, and mitigating fatigue-related safety risks in safety-sensitive occupations. Unlike prescriptive duty-hour regulations, FRMS adopt a risk-based framework that integrates continuous monitoring of sleep opportunities, work schedules, workload characteristics, and fatigue indicators at both individual and organizational levels. Although direct empirical evaluation of FRMS as a unified system remains limited, substantial evidence supports the effectiveness of its core components—including biomathematical fatigue modeling, fatigue self-reporting, and performance monitoring—in reducing fatigue-related risk and improving injury prevention (7).

The effective construction of FRMS requires strong organizational commitment, workforce engagement, and a supportive safety culture. In healthcare settings, user-centered design approaches have highlighted the importance of aligning fatigue monitoring tools with clinical workflows to ensure acceptability and sustained use among nursing staff (8). Diagnostic frameworks developed through expert consensus further enable organizations to assess FRMS maturity, identify implementation gaps, and prioritize targeted improvements (9). These tools facilitate continuous learning and iterative risk control rather than one-time compliance.

Evidence from other safety-critical industries provides valuable insights for healthcare adaptation. Studies in air traffic control and emergency response contexts indicate that fatigue interacts with workload and cognitive demand, suggesting that FRMS effectiveness depends not only on managing sleep and duty hours but also on optimizing task load, break allocation, and staffing patterns (10, 11). Similarly, applications of biomathematical fatigue models in medical residency programs demonstrate that circadian-informed scheduling can reduce fatigue risk without necessarily reducing total work hours, highlighting the potential of FRMS to enhance safety through smarter work design rather than rigid time restrictions (12).

Emerging technologies, including consumer sleep technologies, offer opportunities for longitudinal fatigue monitoring within FRMS frameworks, although their clinical validity and ethical integration require further evaluation before widespread adoption (13). Importantly, successful FRMS implementation depends on organizational readiness, including leadership support, workforce education, and non-punitive reporting cultures. Hybrid approaches that combine regulatory compliance with risk-based fatigue management have been proposed as pragmatic pathways, particularly in settings with variable resources and safety culture maturity (7).

Overall, FRMS provide a scalable framework for addressing fatigue as a systemic risk embedded within work design. When adapted to nursing environments, FRMS have the potential to support proactive injury prevention by integrating scientific evidence, organizational processes, and continuous safety improvement.

### 3.2 Shift pattern optimization and work time management

Prolonged working hours, rotating night shifts, and insufficient recovery time between shifts are consistently identified as major contributors to nurse fatigue. Empirical studies indicate that extended shifts and circadian misalignment are associated with reduced sleep quality, impaired alertness, and elevated fatigue levels, particularly among nurses engaged in night and rotating shift work (14). In response, evidence-based scheduling strategies emphasize limiting consecutive night shifts, avoiding quick-return intervals shorter than 11 h, and constraining shift duration to reduce cumulative fatigue burden (15).

Participatory and flexible scheduling models have demonstrated additional benefits by enhancing nurses’ perceived control over work time and accommodating individual needs related to age, health status, and family responsibilities (16–18). Such approaches support recovery and may reduce fatigue-related safety risks without compromising staffing adequacy. In parallel, workload-adjusted staffing models that incorporate patient acuity and real-time demand have been developed to optimize nurse allocation and prevent excessive workload accumulation during high-demand periods (19).

Fatigue risk assessment tools, including shift-based indices that account for sleep opportunity and recovery constraints, further enable data-informed scheduling decisions (20). Importantly, quick-return shifts have been repeatedly associated with poor sleep quality and increased work–family conflict, reinforcing the need for minimum rest interval protections within scheduling policies (21). In high-intensity settings such as emergency departments and intensive care units, limiting consecutive long shifts and ensuring protected rest breaks have been associated with reductions in fatigue-related adverse events, including medication errors and needlestick injuries (22, 23).

Collectively, these findings support shift pattern optimization as a foundational component of fatigue risk mitigation. By integrating evidence-based scheduling limits, participatory planning, and workload-responsive staffing, healthcare organizations can reduce fatigue exposure while maintaining care continuity and patient safety.

### 3.3 Implementation of protective rest and strategic napping

Protective rest periods embedded within work schedules are essential for mitigating fatigue during high-intensity nursing shifts. Insufficient rest during duty hours has been linked to declines in vigilance and clinical judgment, increasing the likelihood of errors and injuries (24). Strategic napping—typically short naps of 10–30 min taken during extended or night shifts—has been shown to improve alertness, cognitive performance, and motor function, particularly under conditions of circadian disruption (25).

The effectiveness of protective rest and napping depends on organizational policies that ensure coverage continuity and legitimize rest as a safety practice rather than an individual concession. Without institutional support, nurses may be reluctant or unable to utilize rest opportunities due to workload pressure or cultural stigma (26). Accordingly, successful implementation requires coordinated staffing plans, designated rest spaces, and leadership endorsement.

In fatigue-prone care environments, protected rest and strategic napping function as organizational risk controls that complement scheduling optimization and staffing strategies. When systematically integrated into work design, these interventions contribute to both injury prevention and sustained performance under demanding clinical conditions.

### 3.4 Staffing and patient acuity matching

Aligning nurse staffing levels with patient acuity and care complexity is a critical strategy for preventing fatigue-related harm. Evidence indicates that inadequate staffing relative to patient needs contributes to excessive workload, fatigue accumulation, and compromised injury prevention. Studies across psychiatric, acute care, and intensive care settings demonstrate that staffing models accounting for patient dependency, turnover, and clinical complexity are associated with reductions in adverse events, including inpatient falls, missed care processes, and healthcare-associated infections (27–32).

Validated acuity assessment tools enable more precise matching of nursing resources to patient needs and support dynamic staffing adjustments during demand surges. During periods of heightened workload variability, such as public health emergencies, maintaining sufficient baseline staffing has been shown to be more protective and cost-effective than reliance on temporary workforce expansion alone (30). By preventing sustained overload, acuity-responsive staffing mitigates fatigue accumulation and supports safer care delivery.

### 3.5 Cross-regional and cross-institutional experiences in shift scheduling management

Shift scheduling and fatigue management practices vary widely across healthcare systems, shaped by resource availability, technological capacity, and cultural context. In resource-rich settings, intelligent scheduling platforms and decision-support systems have been increasingly adopted to optimize shift allocation and reduce administrative burden, with mixed but generally favorable effects on staff satisfaction and workload balance (33, 34). In contrast, resource-limited environments often rely on simplified scheduling rules, shorter fixed shifts, and intensified workforce training to manage fatigue under constrained conditions (35, 36).

Cultural norms and institutional attitudes toward shift work and fatigue further influence the acceptance and effectiveness of scheduling interventions. Variations in compliance with working time regulations and perceptions of fatigue-related risk highlight the need for context-sensitive implementation strategies (37–39). Overall, cross-regional comparisons underscore that effective fatigue management requires adaptable scheduling approaches aligned with local resources, workforce characteristics, and organizational culture rather than uniform solutions.

## 4 Engineering and ergonomic solutions

### 4.1 Safe patient handling programs and assistive devices

Safe patient handling programs constitute a cornerstone of injury prevention in nursing environments, particularly for reducing musculoskeletal disorders associated with patient transfers and repositioning. These programs typically combine standardized handling protocols, workforce training, and the systematic use of assistive devices to minimize physical strain. Biomechanical and observational studies consistently demonstrate that mechanical aids—such as ceiling lifts, air-assisted transfer systems, and powered transfer devices—reduce spinal loading, muscle activation, and perceived exertion during patient handling tasks (40–43).

Among available technologies, powered and air-assisted devices have shown particular effectiveness in lowering physical workload and improving caregiver safety, while also facilitating controlled patient movement. Emerging innovations, including back-support exoskeletons, further aim to offload lumbar stress and reduce cumulative musculoskeletal injury risk. Quantitative assessment tools, such as biomechanical load estimation models, have supported their potential protective effects, although usability and workflow integration remain critical determinants of adoption (40).

Despite robust evidence supporting their efficacy, assistive devices are frequently underutilized in clinical practice. Barriers include limited device availability, insufficient training, time constraints, and perceptions of workflow disruption (44). Consequently, safe patient handling programs must extend beyond equipment provision to include education, maintenance, leadership support, and organizational policies that normalize device use as a standard safety practice.

### 4.2 Spatial layout and environmental optimization

Optimizing the spatial layout of nursing units is an effective strategy to reduce unnecessary movement, physical workload, and environmental hazards that contribute to fatigue-related injuries. Inefficient layouts increase walking distances, awkward postures, and task interruptions, which collectively elevate the risk of slips, trips, collisions, and musculoskeletal strain. Empirical studies in inpatient units demonstrate that proximity between nurse stations, patient rooms, medication areas, and supply rooms is associated with improved workflow efficiency and reduced physical burden among nursing staff (45).

Ergonomic redesign approaches, including participatory ergonomics and digital human modeling, have been shown to reduce ergonomic risk scores without substantial financial investment (46). Environmental factors such as floor material selection and lighting design also play a critical role in injury prevention. Slip-resistant flooring reduces fall risk in wet or high-traffic areas, while appropriate lighting mitigates visual fatigue and supports sustained attention during safety-critical tasks (47).

Beyond physical safety, optimized spatial environments facilitate communication, visibility, and teamwork, indirectly supporting safety culture and fatigue mitigation (48, 49). Integrating ergonomic layout design with broader organizational safety initiatives enhances both injury prevention and care efficiency in fatigue-prone nursing settings.

### 4.3 Sharp safety technologies and protective measures

Sharps injuries remain a persistent occupational hazard for healthcare workers, particularly in fatigue-prone clinical environments. Safety-engineered devices (SEDs), including auto-retractable needles and shielded sharps, have been widely introduced to reduce injury risk. Evidence indicates that increased availability and routine use of SEDs are associated with reductions in sharps injury incidence; however, incomplete adoption and improper use continue to limit their effectiveness (50, 51).

Effective sharps injury prevention also depends on robust waste management systems. Inadequate disposal practices, overfilled containers, and delayed replacement significantly increase injury risk (52, 53). Accordingly, comprehensive prevention strategies must integrate device technology with accessible disposal infrastructure, clear protocols, and timely maintenance.

Training and safety culture reinforcement are essential complements to technological solutions. Studies consistently report underreporting of sharps injuries and inconsistent adherence to safety protocols, particularly under conditions of high workload and fatigue (54–56). Non-punitive reporting systems, prompt access to postexposure prophylaxis, and ongoing education are critical to mitigating both physical and psychological consequences of sharps injuries (57).

### 4.4 Personal protective equipment and comfort-oriented design

Personal protective equipment (PPE) plays a vital role in safeguarding healthcare workers but may also contribute to fatigue and discomfort when poorly designed or used for prolonged periods. Studies conducted during the COVID-19 pandemic revealed that extended PPE use is associated with thermal discomfort, increased physical strain, impaired communication, and reduced task performance among nurses and clinicians (58, 59).

Human factors and ergonomic design principles have been increasingly applied to improve PPE comfort without compromising protective efficacy. Innovations addressing fit, weight distribution, ventilation, and anti-fogging have been shown to enhance usability and reduce fatigue-related burden (60). Advanced materials, including phase change materials and customized respirator designs, further support thermal regulation and prolonged wear comfort (61, 62).

Importantly, comfort influences compliance. Low perceived comfort has been identified as a barrier to consistent PPE use, underscoring the need to integrate ergonomic considerations into procurement and implementation decisions (63). PPE design that minimizes physical and cognitive burden contributes not only to individual well-being but also to overall safety culture in fatigue-prone environments.

### 4.5 Technological innovations and intelligent assistive tools

The technologies discussed in this section are included based on their direct relevance to mitigating fatigue-related injury mechanisms—namely, physical overload, cognitive strain, and alertness failures—rather than as a comprehensive review of all healthcare technologies. Emerging technologies offer new opportunities to mitigate fatigue-related injury risk through real-time monitoring, automation, and intelligent decision support. Wearable sensors and electronic skin technologies enable continuous assessment of physical workload, posture, and thermal strain, supporting early identification of fatigue-related risk states (64). When integrated with machine learning algorithms, these systems may facilitate proactive interventions before injury occurs.

Robotic assistance for patient handling and material transport has been explored as a strategy to directly reduce physical workload—a key pathway linking fatigue to musculoskeletal injury risk—particularly in high-demand care settings (65).

Alarm management technologies represent another critical area of innovation. Excessive and non-actionable alarms contribute to cognitive overload and alarm fatigue, undermining both staff and patient safety (66). Intelligent alarm systems that prioritize clinically relevant alerts and filter false alarms have shown promise in reducing information overload and supporting sustained attention in critical care environments (67–71).

Overall, technological innovations should be viewed as supportive tools within broader fatigue risk management frameworks rather than standalone solutions. Their effectiveness depends on human-centered design, organizational readiness, and alignment with safety culture priorities.

## 5 Behavior and team practices

### 5.1 Microbreaks and fatigue self-regulation behaviors

Microbreaks—brief, self-initiated rest periods taken during work—have emerged as an effective behavioral strategy for mitigating localized physical fatigue and sustaining cognitive performance in high-demand healthcare environments. Evidence grounded in self-regulatory resource theory indicates that microbreaks function as short recovery episodes that replenish attentional and physical resources, particularly among individuals experiencing insufficient recovery outside work (72).

In clinical and procedural settings, microbreak interventions incorporating brief stretching or posture adjustments have been associated with reductions in musculoskeletal discomfort and perceived fatigue without prolonging task duration (73, 74). Beyond physical relief, microbreaks support cognitive recovery by interrupting sustained attentional demands, thereby preserving vigilance and decision-making capacity during safety-critical tasks (75). These effects are especially relevant in nursing contexts characterized by prolonged standing, repetitive movements, and continuous multitasking.

The effectiveness of microbreaks depends largely on behavioral autonomy and a supportive safety climate. Nurses are more likely to engage in fatigue self-regulation behaviors when microbreaks are perceived as legitimate safety practices rather than signs of reduced commitment (76). Barriers such as normalized fatigue culture, high workload, and limited managerial support may inhibit microbreak utilization, underscoring the importance of aligning individual behaviors with organizational safety values (77).

Overall, microbreaks represent a low-cost, behaviorally driven fatigue mitigation strategy that complements organizational rest policies. When embedded within a positive safety culture, they support sustained performance and reduce fatigue-related injury risk at the individual and team levels.

### 5.2 Fatigue awareness communication and team collaboration

Effective communication and team collaboration are essential for identifying and managing fatigue-related risks in nursing environments. Open fatigue awareness communication—where nurses can disclose fatigue status without fear of stigma—enhances shared situational awareness and enables teams to adapt task allocation and supervision accordingly. Non-technical skills, including communication, teamwork, and stress management, have been consistently associated with improved performance and guideline adherence in high-risk clinical settings (78).

Integrating fatigue considerations into routine team briefings, handovers, and safety huddles further strengthens collective risk management. Multidisciplinary training programs emphasize that acknowledging clinician fatigue is integral to patient safety rather than a personal limitation (79, 80). During periods of sustained stress, such as public health emergencies, leadership communication characterized by visibility, transparency, and emotional support has been shown to mitigate fatigue and reinforce team cohesion (81).

Team-level factors such as trust, shared mental models, and psychological safety are inversely associated with fatigue and burnout among nurses (82, 83). Technological tools that facilitate fatigue monitoring and information sharing may further support communication, provided they are integrated with clear protocols and do not exacerbate cognitive burden (84, 85).

By normalizing fatigue discussions and strengthening collaboration, healthcare teams can implement compensatory strategies that buffer the impact of fatigue on performance and safety.

### 5.3 Training for high-risk tasks under fatigue conditions

Targeted training for high-risk nursing tasks, such as patient handling and sharps use, is critical in fatigue-prone care environments. Training programs that emphasize not only procedural skills but also the rationale underlying safe practices enhance risk recognition and adaptive decision-making under fatigue conditions (86, 87). Participatory and simulation-based approaches encourage reflective learning and improve preparedness for real-world complexity.

Fatigue impairs cognitive processing, motor coordination, and situational awareness, increasing vulnerability during safety-critical tasks. Accordingly, incorporating fatigue awareness into training curricula has been recommended to enhance compliance and error prevention (88). Simulation-based and hybrid training modalities have demonstrated effectiveness in improving skill retention, confidence, and non-technical competencies, particularly in high-acuity contexts (89).

Human factors education further sensitizes nurses to how fatigue alters perception and performance, reinforcing the importance of system safeguards and teamwork during high-risk activities (90). Collectively, fatigue-informed training supports safer task execution and complements engineering and organizational interventions.

### 5.4 Fatigue-related incident reporting and feedback mechanisms

Non-punitive reporting systems for fatigue-related incidents and near-misses are fundamental to learning-oriented safety management. When nurses are encouraged to report fatigue-associated errors without fear of blame, organizations gain critical insights into systemic vulnerabilities rather than attributing failures to individual shortcomings. Punitive cultures, by contrast, discourage reporting and obscure the role of fatigue as an occupational hazard.

Data derived from fatigue-related incident reports enable organizations to identify patterns, refine workflows, and target interventions addressing scheduling, staffing, and environmental contributors to fatigue. Effective systems close the feedback loop by communicating findings and corrective actions to frontline staff, reinforcing trust and engagement.

Framing fatigue as a system-level risk aligns with human factors engineering principles and supports continuous improvement. Transparent reporting and feedback mechanisms thus play a pivotal role in preventing fatigue-related harm and strengthening organizational resilience (91–93).

**Figure 4:**
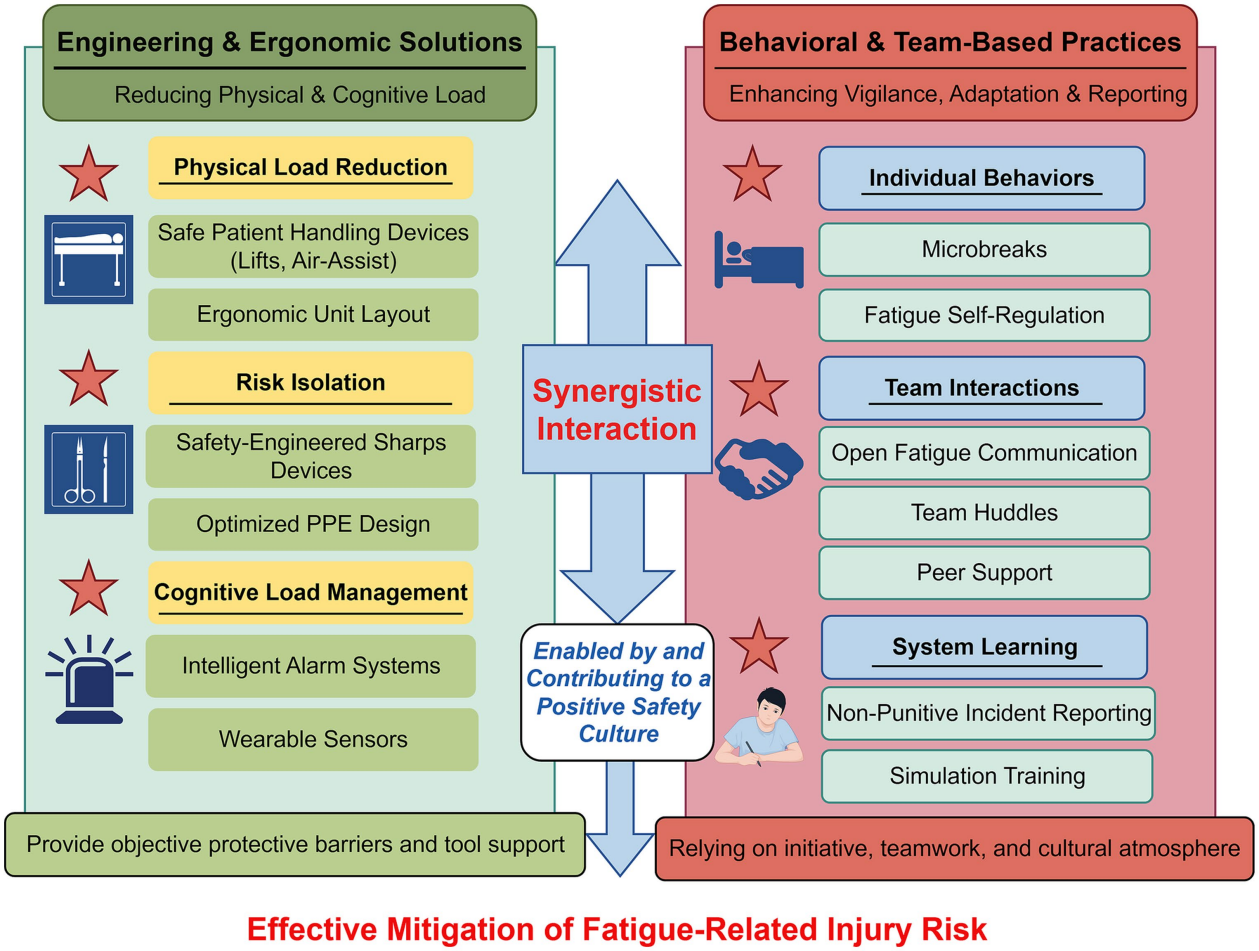
Synergistic interaction between engineering controls and behavioral practices. This model illustrates how engineering solutions (environmental redesign, technology) and behavioral practices (individual and team actions) function as complementary pillars, with safety culture serving as the catalytic enabler that enhances their combined effectiveness. Figure drawn by Figdraw.com.

As detailed in the preceding sections, sustainable injury prevention requires the convergence of environmental redesign and human action. Figure 4 synthesizes this interplay, depicting how engineering controls and behavioral practices function as complementary pillars, whose synergy is catalyzed by a positive safety culture to effectively mitigate injury risk.

### 5.5 Psychological support and occupational health promotion

Psychological support and occupational health promotion are essential for mitigating the cumulative mental and physical effects of fatigue in nursing environments. Chronic fatigue is closely linked to anxiety, depression, burnout, and compassion fatigue, particularly under conditions of high workload and prolonged stress exposure (94). Psychological support interventions, including counseling and peer support, have demonstrated effectiveness in reducing distress, absenteeism, and emotional exhaustion among healthcare workers (95).

Protective psychosocial factors such as resilience, social support, and family-supportive leadership behaviors buffer the impact of fatigue on mental health (96–98). Sleep quality, a central determinant of fatigue vulnerability, is frequently compromised among nurses exposed to extended shifts and occupational stress, highlighting the importance of sleep hygiene promotion and recovery-focused scheduling (97, 99).

Organizational empowerment and supportive safety culture further contribute to fatigue mitigation by enhancing work motivation and psychological capital (100, 101). In high-stress contexts, sustained access to mental health resources and stigma-free help-seeking pathways are critical for maintaining workforce well-being and preventing fatigue-related attrition (102–104).

The evidence-based interventions discussed across the organizational, engineering, and behavioral domains are synthesized in Table 1, which provides a comparative overview of their mechanisms, examples, and supporting references.

## 6 Implementation barriers and effect evaluation

### 6.1 Resource constraints and cost-effectiveness considerations

Resource constraints constitute a major barrier to the implementation of fatigue management and injury prevention strategies in healthcare settings. These constraints extend beyond financial limitations to include shortages in staffing, training capacity, time, and infrastructure. The COVID-19 pandemic served as a profound stress test, exposing the fragility of existing fatigue management systems (105). Key lessons learned include the critical importance of surge capacity planning that integrates fatigue risk assessment, not just staff numbers. The pandemic underscored that in crisis situations, flexible, risk-based approaches, such as simplified scheduling rules and enhanced mental health support, are as vital as rigid regulations. It also highlighted the need for resilient infrastructure, including adequate rest spaces and readily available PPE that does not compromise comfort, to sustain workforce health during prolonged periods of high demand. Future preparedness efforts must embed these pandemic lessons into permanent, scalable fatigue management frameworks.

In response, resource-efficient interventions have gained attention. In-situ simulation training has emerged as a pragmatic approach to enhance both technical and non-technical competencies without the need for dedicated simulation facilities, demonstrating feasibility in high-demand clinical contexts (105). Similarly, low-cost digital interventions, such as brief well-being messaging and virtual training platforms, have shown potential to support workforce resilience and education at scale (106, 107).

Economic evaluation plays a critical role in prioritizing interventions under resource constraints. Evidence from healthcare and public health indicates that targeted prevention programs can be cost-effective or cost-saving when compared with standard practice, particularly when they reduce injury-related absenteeism, turnover, and downstream healthcare costs (108, 109). Importantly, human resource availability often represents a greater limiting factor than budget alone, underscoring the need for integrated workforce planning alongside financial investment (110).

Economic evaluation plays a critical role in prioritizing interventions under resource constraints. Evidence specifically relevant to fatigue-related injury prevention in nursing populations demonstrates the potential for cost savings through targeted interventions. For example, Olinski and Norton described how a safe patient handling program in a multi-hospital system—an intervention directly targeting musculoskeletal injury risk in fatigue-prone nurses—yielded an 82% reduction in OSHA recordable patient handling injuries and over 80% reduction in workers’ compensation costs (111). These savings reflect reduced injury-related absenteeism and claim expenses, both of which are exacerbated by fatigue-induced physical strain during patient transfers.

Similarly, interventions that reduce cognitive load and alertness failures—key mechanisms linking fatigue to injury—have demonstrated economic benefits. Colpaert et al. found that implementing computer-assisted medication prescribing in intensive care units significantly reduced medication error incidence and severity, with projected annual cost savings (112). While this intervention primarily targets patient safety, the mechanism involves reducing cognitive burden on nurses during high-fatigue periods, thereby also potentially reducing occupational injury risk associated with error recovery and task reprioritization.

More broadly, research indicates that implementing fatigue risk management systems in healthcare settings can yield cost savings through prevention of medical errors, reduced absenteeism, and decreased staff turnover (113, 114). These economic returns stem from addressing fatigue as a root cause of multiple adverse outcomes rather than as an isolated individual concern.

Overall, implementation strategies must balance effectiveness with feasibility, prioritizing interventions that deliver meaningful safety benefits while remaining adaptable to varying resource contexts.

### 6.2 Alarm burden and information overload

Alarm fatigue represents a prominent and persistent implementation challenge in fatigue-prone nursing environments. Excessive alarm exposure, particularly from non-actionable or false alarms, contributes to cognitive overload, desensitization, and delayed responses, thereby increasing the risk of both staff injury and patient harm (115). Studies in intensive care settings consistently demonstrate associations between alarm burden, perceived role overload, and error proneness among nurses.

Technological solutions aimed at alarm optimization—including intelligent filtering, prioritization algorithms, and context-aware alerting systems—have shown promise in reducing alarm frequency and improving signal relevance (67). However, alarm fatigue is not solely a technical issue. Fragmented device ecosystems, inconsistent alarm design, and poor integration with clinical workflows further exacerbate information overload (69, 116).

Effective alarm management requires a systems-level approach combining human factors engineering, clinician education, and organizational governance. Establishing shared mental models around alarm relevance and embedding alarm metrics into quality improvement processes are essential to sustaining implementation gains (117). Without such integration, even well-designed technologies may fail to achieve lasting impact.

### 6.3 Safety culture deficiencies and their impact

Deficiencies in safety culture pose significant barriers to the implementation of fatigue mitigation strategies. In organizations where punitive norms, hierarchical barriers, or stigma surrounding fatigue persist, nurses may be reluctant to report fatigue-related concerns or incidents, resulting in underrecognition of systemic risks (118, 119). Such environments often frame fatigue as an individual weakness rather than an occupational hazard, undermining prevention efforts.

Leadership engagement and psychological safety are central determinants of safety culture maturity. Evidence indicates that visible leadership commitment, balanced accountability, and continuous feedback mechanisms facilitate fatigue reporting and adherence to safety practices (120, 121). Conversely, weak safety culture is associated with poor protocol compliance and limited learning from adverse events, perpetuating fatigue-related harm (122, 123).

Addressing safety culture deficiencies requires sustained organizational investment in leadership development, staff engagement, and education that normalizes fatigue as a legitimate safety concern rather than a personal failure.

The translation of strategies into practice is hindered by a constellation of interrelated barriers. Tables 2, 3 summarizes these major implementation challenges and the concomitant difficulties in evaluating intervention effectiveness.

### 6.4 Evaluation metrics and data collection challenges

Evaluating fatigue-related injury prevention interventions requires multidimensional outcome metrics that capture clinical, organizational, and human factors domains. Commonly used indicators include injury incidence rates, near-miss reporting frequency, sleep quality measures, and workforce retention metrics, each reflecting different aspects of intervention effectiveness.

However, the validity of these metrics is frequently compromised by data collection challenges, including underreporting, inconsistent event definitions, and reliance on subjective self-reports. Near-miss events, in particular, are prone to underdocumentation due to fear of blame or reporting fatigue. Standardized data collection protocols, validated measurement instruments, and integration of reporting systems into routine workflows are essential to improving data completeness and accuracy.

Technological solutions such as electronic incident reporting platforms and automated data capture may reduce reporting burden and enhance surveillance. Equally important is leadership support for transparent reporting and feedback, which reinforces data quality and sustains engagement. Robust evaluation frameworks are critical for generating reliable evidence to guide fatigue mitigation strategies and continuous improvement (124–127).

### 6.5 Adaptation to regional and institutional differences

Fatigue-related injury prevention strategies must be adapted to regional, cultural, and institutional contexts. Variations in resource availability, workforce composition, regulatory frameworks, and safety culture influence both fatigue exposure and the feasibility of interventions. Global health data highlight substantial heterogeneity in health outcomes and system capacity across regions, underscoring the limitations of uniform implementation models (128).

In resource-limited settings, low-cost interventions such as optimized scheduling, peer support, and fatigue awareness training may offer the greatest impact. In contrast, resource-rich environments may leverage advanced monitoring technologies and ergonomic redesign. Even within the same country, institutional differences in staffing, leadership, and organizational culture necessitate tailored approaches (129).

Cross-institutional collaboration and knowledge sharing facilitate adaptation by enabling organizations to learn from diverse implementation experiences. Flexible, context-sensitive strategies are therefore essential for achieving sustainable fatigue mitigation across heterogeneous healthcare systems (129–132).

### 6.6 Toward a standardized core outcomes bundle for fatigue-related injury prevention

The heterogeneity in outcome measures observed across the reviewed literature—ranging from occupational injury incidence to patient safety events and subjective fatigue ratings—limits cross-study comparability and impedes the development of evidence-based benchmarks. Based on the synthesized evidence, we propose a minimum core outcomes bundle for institutions implementing fatigue-related injury prevention strategies. This bundle is intended to enhance data standardization while remaining feasible across diverse healthcare settings.

Recommended core outcomes:

1. Recordable occupational injury rates: Including OSHA-recordable injuries (or equivalent national definitions) stratified by injury type (e.g., needlestick/sharps, slips/trips/falls, patient handling-related MSDs). Recommended measurement periodicity: Quarterly surveillance with annual summary.
2. Near-miss reporting rates: Capturing events that could have resulted in injury but did not, as an indicator of safety climate and early warning. Recommended periodicity: Monthly tracking with quarterly review.
3. Validated fatigue measure: A standardized instrument such as the Occupational Fatigue Exhaustion Recovery (OFER) scale, Chalder Fatigue Questionnaire, or single-item fatigue severity ratings. Recommended periodicity: Cross-sectional surveys at baseline and annually, with optional real-time sampling in high-risk units.
4. Sleep opportunity or duration metrics: Objective or self-reported total sleep time per 24-h period, particularly relevant for shift-working nurses. Recommended periodicity: Rolling sampling or annual survey.
5. Workload indicators: Staffing ratios (patient-to-nurse) and, where available, acuity-adjusted workload measures. Recommended periodicity: Continuous administrative data with annual summary.
6. Workforce stability indicators: Turnover rates, absenteeism rates (sick leave), and intent to leave. Recommended periodicity: Quarterly HR data, annual survey.
7. Patient safety indicators (context-dependent): Where relevant to institutional priorities, selected patient injury prevention linked to fatigue (e.g., medication errors, inpatient falls) may be included, with clear designation as patient safety rather than occupational injury outcomes.

Institutions are encouraged to adopt this bundle incrementally, prioritizing measures aligned with existing data infrastructure. Future research should focus on validating minimum data set standards and establishing benchmark ranges across healthcare settings.

### 6.7 The reporting paradox: safety culture and incident rates

An important methodological consideration that warrants explicit acknowledgment is the potential for reporting bias to distort observed intervention effects, particularly during early implementation phases. The manuscript has emphasized the importance of non-punitive safety cultures and robust reporting systems for fatigue-related incidents and near-misses. However, improvements in safety climate—while essential for long-term risk reduction—may initially increase reported incident and near-miss rates as psychological safety improves and reporting barriers diminish (91–93). This phenomenon creates a paradoxical observation period during which interventions targeting safety culture may appear to increase adverse event rates, potentially leading to premature discontinuation of effective programs if misinterpreted.

This reporting paradox is particularly relevant to the evaluation framework proposed in Section 6.6. Institutions implementing fatigue-related injury prevention strategies should anticipate a potential short-term increase in reported events following safety culture interventions and should interpret such increases as indicators of improved detection and reporting rather than deteriorating safety performance (118, 120). To mitigate misinterpretation, evaluators should:

1. Establish baseline reporting rates prior to intervention implementation;
2. Track both reported incidents and independent safety indicators (e.g., observational audits, objective injury data) to distinguish true safety changes from reporting changes;
3. Communicate the expected reporting paradox to stakeholders during program rollout; and.
4. Allow sufficient follow-up time (minimum 12–24 months) for reporting stabilization before drawing conclusions about intervention effectiveness.

Failure to account for this reporting bias may lead to underestimation of intervention benefits and undermine sustained investment in fatigue mitigation strategies.

## 7 Future research and development directions

To advance the field, targeted research and development are needed across several key areas. Table 4 outlines these priority domains, linking them to critical research questions and their potential impact on practice and policy.

### 7.1 Development of multidisciplinary collaboration models

Addressing fatigue-related injury risk in nursing environments requires multidisciplinary collaboration that integrates expertise from occupational health, ergonomics, sleep medicine, behavioral science, and healthcare management. Fatigue is a complex, multifactorial phenomenon embedded within work systems, and no single discipline can adequately address its physiological, cognitive, and organizational dimensions in isolation.

Evidence from healthcare and other safety-sensitive sectors demonstrates that multidisciplinary teams enhance situational awareness, communication, and coordinated risk management. In clinical settings, nurse-led initiatives and interprofessional safety briefings have been associated with improved teamwork, reduced delays in care, and enhanced adherence to safety practices (133–136). Simulation-based multidisciplinary training further supports shared mental models and collective preparedness for high-risk situations (137).

To be effective, collaboration models must be supported by clear role delineation, leadership engagement, and continuous evaluation. Multidisciplinary frameworks provide a foundation for translating scientific evidence into practical, context-sensitive interventions that address fatigue as a system-level safety challenge.

### 7.2 Application and validation of emerging technologies

Emerging technologies—including wearable sensors, artificial intelligence, and big data analytics—offer promising tools for monitoring fatigue and predicting injury risk in healthcare settings. Wearable technologies enable continuous assessment of biomechanical load, physiological strain, and recovery patterns, supporting early identification of fatigue-related risk states (138). When combined with machine learning algorithms, these data streams may inform adaptive scheduling and workload management. Despite their potential, the clinical application of these technologies remains limited by challenges related to data accuracy, interoperability, user acceptance, and ecological validity. Evidence from healthcare technology implementation highlights the importance of human-centered design and workflow integration to prevent unintended consequences such as alert fatigue or increased cognitive burden (139, 140).

Future research should prioritize rigorous field validation, including pragmatic trials and implementation studies, to evaluate not only technical performance but also impact on injury prevention, usability, and organizational processes. Such evidence is essential before widespread adoption in fatigue-prone nursing environments.

### 7.3 Development of personalized fatigue management strategies

Fatigue manifests heterogeneously across individuals due to differences in circadian rhythms, health status, psychological traits, and recovery capacity. Accordingly, personalized fatigue management strategies are increasingly recognized as a necessary complement to standardized organizational interventions. Chronobiology-informed scheduling and biomathematical modeling have demonstrated potential to account for individual variability in fatigue vulnerability and recovery (7, 141). Digital health tools and mobile applications offer scalable platforms for personalized fatigue self-management by integrating sleep, activity, and symptom data (142–144). Participatory design approaches that engage end users in tool development enhance acceptability and contextual relevance, as demonstrated in fatigue management programs for chronic conditions and post-viral syndromes (145). In occupational settings, personalized approaches should be integrated within organizational fatigue risk management frameworks to avoid shifting responsibility solely to individuals. Future research should examine how personalized and system-level strategies can be aligned to maximize safety and equity.

### 7.4 Safety culture development and behavior change research

Safety culture fundamentally shapes how fatigue is perceived, reported, and managed within healthcare organizations. Variability in safety culture across professional groups and hierarchical levels influences reporting behaviors and intervention uptake (146). Disruptive behaviors and psychological unsafety undermine communication and teamwork, exacerbating fatigue-related risk (147). Behavior change research offers structured frameworks, such as capability–opportunity–motivation models, to guide the design of fatigue mitigation interventions (148). Participatory education, leadership training, and experiential learning approaches—including simulation and virtual reality—have demonstrated effectiveness in enhancing safety awareness and proactive behaviors (149–153). Future research should focus on identifying mechanisms through which leadership behaviors, psychological safety, and organizational norms interact to influence fatigue-related behaviors. Longitudinal studies are particularly needed to assess the sustainability of safety culture interventions in fatigue-prone care environments.

### 7.5 Economic evaluation and policy support

Economic evaluation is essential for informing policy decisions and sustaining fatigue management interventions. While fatigue mitigation strategies—such as regulated work hours, protected rest, and ergonomic redesign—require upfront investment, evidence suggests that they may reduce long-term costs associated with injury, absenteeism, turnover, and compromised care quality (154).

Policy support plays a critical role in institutionalizing fatigue risk management. Regulatory frameworks that mandate rest periods, support non-punitive reporting, and promote safe work design provide structural incentives for organizational compliance (155). However, implementation barriers persist, particularly where fatigue management practices conflict with professional norms or operational pressures (156, 157).

Integrating economic evidence with human factors and organizational research can support the development of policies that are both effective and feasible. Such alignment is necessary to embed fatigue management within healthcare systems as a core component of occupational health and patient safety.

### 7.6 Limitations of the reviewed evidence

The findings of this review should be interpreted in light of several limitations within the existing evidence base. First, the majority of studies are observational or quasi-experimental, limiting causal inferences about intervention effectiveness. Second, substantial heterogeneity exists in outcome measures (e.g., self-reported fatigue vs. objective injury data), making cross-study comparisons difficult. Third, many interventions are studied in single-center or resource-rich settings, raising questions about generalizability to diverse healthcare contexts. Fourth, publication bias may lead to an overrepresentation of positive findings. Finally, the rapid evolution of technologies, such as AI-driven monitoring, outpaces the availability of rigorous longitudinal evaluation.

## 8 Conclusion

Drawing upon the synthesized evidence, Table 5 consolidates actionable, integrated recommendations for stakeholders at the organizational, team, and policy levels, providing a roadmap for implementing comprehensive fatigue-related injury prevention.

Nurse fatigue represents a complex occupational health challenge driven by the interaction of organizational demands, physical work conditions, and behavioral factors, with substantial implications for injury risk and patient safety. Evidence synthesized in this review underscores that fatigue-related harm cannot be effectively mitigated through isolated interventions. Instead, injury prevention in fatigue-prone nursing environments requires an integrated, system-oriented approach that aligns work design, engineering controls, and team-based practices.

Organizational strategies such as optimized scheduling, fatigue risk management systems, and acuity-responsive staffing establish foundational protections against fatigue accumulation. Engineering and ergonomic interventions—including assistive patient handling technologies, environmental optimization, and intelligent alarm management—reduce physical and cognitive load. Behavioral and team-based practices, supported by a positive safety culture, enable frontline nurses to recognize and manage fatigue proactively.

However, the translation of evidence into practice remains constrained by resource limitations, cultural barriers, and data challenges. Addressing these barriers requires context-sensitive implementation strategies, robust evaluation frameworks, and sustained leadership commitment. Future progress depends on multidisciplinary collaboration, validation of emerging technologies, and the integration of personalized fatigue management within organizational systems.

Ultimately, embedding fatigue prevention within healthcare policy and safety culture is essential not only for protecting nurses’ health but also for sustaining workforce stability and care quality. As healthcare systems continue to face increasing demands, comprehensive and adaptive fatigue mitigation strategies are indispensable for building safer and more resilient nursing environments.

## References

[1] Yang Y Li J Wang H Liu Y Wang X Yuan S. Factors contributing to muscle fatigue of low back region in ICU nurses: a qualitative study. Nurs Open. (2025) 12:e70146. doi: 10.1002/nop2.70146, 40055933 PMC11889409

[2] Li M Huo L Du F Li W Zhang H Shi B. Prevalence, emotional and follow-up burden of insulin injection-related needle-stick injuries among clinical nurses in Shaanxi Province, west of China: a cross-sectional study. Nurs Open. (2022) 9:1984–94. doi: 10.1002/nop2.1200, 35343081 PMC9190700

[3] Kincl L Syron L Lucas D Vaughan A Bovbjerg V. Relationship of personal, situational, and environmental factors to injury experience in commercial fishing. J Saf Res. (2023) 87:375–81. doi: 10.1016/j.jsr.2023.08.009, 38081709 PMC10807482

[4] Zeng Z Zhou S Liu M. Research progress on assessment tools related to occupational fatigue in nurses: a traditional review. Front Public Health. (2024) 12:1508071. doi: 10.3389/fpubh.2024.1508071, 39712300 PMC11659218

[5] Czernecki K Nowicki G Graczyk M Ślusarska B. Fatigue of palliative care nursing staff and selected sociodemographic, occupational and cognitive predictors: a cross-sectional study. Int J Occup Med Environ Health. (2025) 38:41–56. doi: 10.13075/ijomeh.1896.02520, 39912639 PMC11952196

[6] Caboral-Stevens M Raymond D Evangelista LS. Well-being, occupational fatigue, and sleep quality among Filipino nurses working during COVID-19. Asia J Nurs Educ Res. (2023) 13:67–72. doi: 10.52711/2349-2996.2023.00016, 37581171 PMC10425155

[7] Sprajcer M Thomas MJW Sargent C Crowther ME Boivin DB Wong IS. How effective are fatigue risk management systems (FRMS)? A review. Accid Anal Prev. (2022) 165:106398. doi: 10.1016/j.aap.2021.106398, 34756484 PMC8806333

[8] Steege LM Arsenault Knudsen ÉN Brzozowski S Cho H. Addressing occupational fatigue in nurses: a user-centered design approach for fatigue risk management. J Nurs Adm. (2022) 52:167–76. doi: 10.1097/NNA.0000000000001125, 35179143

[9] Maisey G Cattani M Devine A Dunican IC. Fatigue risk management systems diagnostic tool: validation of an organizational assessment tool for shift work organizations. Saf Health Work. (2022) 13:408–14. doi: 10.1016/j.shaw.2022.08.002, 36579003 PMC9772465

[10] Li WC Kearney P Zhang J Hsu YL Braithwaite G. The analysis of occurrences associated with air traffic volume and air traffic controllers’ alertness for fatigue risk management. Risk Anal. (2021) 41:1004–18. doi: 10.1111/risa.13594, 32920882

[11] Fletcher A Stewart S Heathcote K Page P Dorrian J. Work schedule and seasonal influences on sleep and fatigue in helicopter and fixed-wing aircraft operations in extreme environments. Sci Rep. (2022) 12:8263. doi: 10.1038/s41598-022-08996-2, 35585079 PMC9117332

[12] Schwartz LP Devine JK Hursh SR Davis JE Smith M Boyle L. Addressing fatigue in medical residents with biomathematical fatigue modeling. J Occup Health. (2021) 63:e12267. doi: 10.1002/1348-9585.12267, 34390073 PMC8363908

[13] Devine JK Hursh SR. A narrative review on in-flight use of consumer sleep technologies for aviation research. Sleep Adv. (2025) 6:zpaf076. doi: 10.1093/sleepadvances/zpaf076, 41278216 PMC12640197

[14] Yoshida A Asakura K Imamura H Mori S Sugimoto M Michikawa T . Relationship between working hours and sleep quality with consideration to effect modification by work style: a community-based cross-sectional study. Environ Health Prev Med. (2024) 29:19. doi: 10.1265/ehpm.23-00252, 38508769 PMC10965413

[15] Garde AH Begtrup L Bjorvatn B Bonde JP Hansen J Hansen ÅM . How to schedule night shift work in order to reduce health and safety risks. Scand J Work Environ Health. (2020) 46:557–69. doi: 10.5271/sjweh.3920, 32895725 PMC7737811

[16] Epstein M Arakelian E Tucker P Dahlgren A. Managing sustainable working hours within participatory working time scheduling for nurses and assistant nurses: a qualitative interview study with managers and staffing assistants. J Nurs Manag. (2023) 2023:8096034. doi: 10.1155/2023/8096034, 40225667 PMC11918713

[17] Guerriero F Guido R. Modeling a flexible staff scheduling problem in the era of Covid-19. Optim Lett. (2022) 16:1259–79. doi: 10.1007/s11590-021-01776-3, 34276828 PMC8274474

[18] Nakao T Takeishi C Tsutsumi C Sato Y Uchizono Y Shimizu Y. Employment factors associated with daily time management in working people with type 2 diabetes. Jpn J Nurs Sci. (2021) 18:e12395. doi: 10.1111/jjns.12395, 33245208

[19] Behringer W Dodt C. Physician staffing and shift work schedules: concepts for emergency and intensive care medicine. Med Klin Intensivmed Notfmed. (2020) 115:449–57. doi: 10.1007/s00063-020-00722-y, 32840636

[20] Schmidt L Trousselard M Perez C Reynaud E Valero B Schlatter S . High risk, low rest: a new framework for monitoring sleep vulnerability in emergency medicine. Front Public Health. (2025) 13:1679296. doi: 10.3389/fpubh.2025.1679296, 41246078 PMC12615451

[21] Safieh S Shochat T Srulovici E. The mediating role of sleep quality in the relationship between quick-return shift work schedules and work-family conflict: a cross-sectional study. J Nurs Res. (2025) 33:e378. doi: 10.1097/jnr.0000000000000663, 40063001

[22] Imes CC Barthel NJ Chasens ER Dunbar-Jacob J Engberg SJ Feeley CA . Shift work organization on nurse injuries: a scoping review. Int J Nurs Stud. (2023) 138:104395. doi: 10.1016/j.ijnurstu.2022.104395, 36481596

[23] Ayas NT Jeklin AT Tholin H Rogers AE Dodek P Hirsh-Allen AJ . Consecutive nursing shifts and the risk of hypoglycemia in critically ill patients who are receiving intravenous insulin: a multicenter study. J Clin Sleep Med. (2020) 16:949–53. doi: 10.5664/jcsm.8382, 32065114 PMC7849663

[24] Chukwunonso-Ogbu A Fazli SA Kalungi G Malomo O. Sleep deprivation and fatigue in healthcare staff: a clinical audit on the risk to patient safety. Cureus. (2025) 17:e96543. doi: 10.7759/cureus.96543, 41250785 PMC12619980

[25] Ulupınar F Ulupınar S. Effects of shift work on cognitive and motor performance in nurses: a systematic review and meta-analysis. Worldviews Evid-Based Nurs. (2025) 22:e70078. doi: 10.1111/wvn.70078, 41139761

[26] Griffin L Riley R. Exploring the psychological impact of working during COVID-19 on medical and nursing students: a qualitative study. BMJ Open. (2022) 12:e055804. doi: 10.1136/bmjopen-2021-055804, 35738645 PMC9226460

[27] Seeherunwong A Thunyadee C Vanishakije W Thanabodee-Tummajaree P. Staffing and patient-related factors affecting inpatient falls in a psychiatric hospital: a 5-year retrospective matched case-control study. Int J Ment Health Syst. (2022) 16:3. doi: 10.1186/s13033-022-00514-1, 35073938 PMC8787870

[28] MacPhee M Wagner J Udod SA Berry L Perchie G Conway A. Using the synergy tool to determine Regina emergency department staffing needs. Nurs Leadersh (Tor Ont). (2020) 33:29–44. doi: 10.12927/cjnl.2020.26321, 33097103

[29] Ostberg N Ling J Winter SG Som S Vasilakis C Shin AY . Quantifying paediatric intensive care unit staffing levels at a paediatric academic medical Centre: a mixed-methods approach. J Nurs Manag. (2021) 29:2278–87. doi: 10.1111/jonm.13346, 33894027

[30] Griffiths P Saville C Ball JE Jones J Monks T Griffiths P. Beyond ratios - flexible and resilient nurse staffing options to deliver cost-effective hospital care and address staff shortages: a simulation and economic modelling study. Int J Nurs Stud. (2021) 117:103901. doi: 10.1016/j.ijnurstu.2021.103901, 33677251 PMC8220646

[31] Recio-Saucedo A Smith GB Redfern O Maruotti A Griffiths P Smith GB. Observational study of the relationship between nurse staffing levels and compliance with mandatory nutritional assessments in hospital. J Hum Nutr Diet. (2021) 34:679–86. doi: 10.1111/jhn.12847, 33406321

[32] Johnson SS Mietchen MS Lofgren ET. Healthcare worker staffing ratios affect methicillin-resistant *Staphylococcus aureus* acquisition. medRxiv. (2024). doi: 10.1101/2024.02.14.24302485, 38405705 PMC10888980

[33] Nwanaji-Enwerem JC Ehrhardt TF Gordon B Meyer H Cardell A Selby M . Considering burnout and well-being: emergency medicine resident shift scheduling platform and satisfaction insights from a quality improvement project. Healthcare (Basel). (2024) 12:612. doi: 10.3390/healthcare12060612, 38540576 PMC10970494

[34] Huang YC Hwang JC Lin YC. The optimization between physician satisfaction and hospital profit in cross-hospital scheduling-a case study of some hospitals in Taiwan. Healthcare. (2021) 9:1004. doi: 10.3390/healthcare9081004, 34442140 PMC8392353

[35] Zhang X Huang DS Guan P. Nursing scheduling mode and experience from the medical teams in aiding Hubei Province during the COVID-19 outbreak: a systematic scoping review of 17 studies. Risk Manag Healthc Policy. (2021) 14:1805–13. doi: 10.2147/RMHP.S302156, 33986617 PMC8110278

[36] Adane A Getnet M Belete M Yeshaw Y Dagnew B. Shift-work sleep disorder among health care workers at public hospitals, the case of Sidama national regional state, Ethiopia: a multicenter cross-sectional study. PLoS One. (2022) 17:e0270480. doi: 10.1371/journal.pone.0270480, 35802698 PMC9269933

[37] Errami I Boutayeb S Errihani H. Burnout among oncology nurses and technicians in Morocco: prevalence, risk factors, and structural equation modeling. Oncoscience. (2025) 12:79–90. doi: 10.18632/oncoscience.623, 40772005 PMC12327364

[38] Çekiç Y Yazgan EÖ Duyan V. Nurses’ experiences, fear of COVID-19, and death anxiety during the COVID-19 pandemic: a cross-sectional study from Turkey. J Psychosoc Nurs Ment Health Serv. (2022) 60:39–48. doi: 10.3928/02793695-20220621-01, 35858188

[39] Sanchez Martinez DA Carrasco Picazo JP Estrella Porter PD Ruiz-Montero R Aginagalde Llorente AH García-Camacho E . Resident physician duty hours, resting times and European working time directive compliance in Spain: a cross-sectional study. Hum Resour Health. (2023) 21:70. doi: 10.1186/s12960-023-00857-x, 37620869 PMC10463816

[40] Fray M Davis KG. Effectiveness of safe patient handling equipment and techniques: a review of biomechanical studies. Hum Factors. (2024) 66:2283–322. doi: 10.1177/00187208231211842, 37947221 PMC11382441

[41] Vinstrup J Jakobsen MD Madeleine P Andersen LL. Biomechanical load during patient transfer with assistive devices: cross-sectional study. Ergonomics. (2020) 63:1164–74. doi: 10.1080/00140139.2020.1764113, 32362200

[42] Law MJJ Ridzwan MIZ Ripin ZM Abd Hamid IJ Law KS Karunagaran J . Evaluation of a motorised patient transfer device based on perceived workload, technology acceptance, and emotional states. Disabil Rehabil Assist Technol. (2024) 19:938–50. doi: 10.1080/17483107.2022.2134472, 36334271

[43] Hwang J Ari H Matoo M Chen J Kim JH. Air-assisted devices reduce biomechanical loading in the low back and upper extremities during patient turning tasks. Appl Ergon. (2020) 87:103121. doi: 10.1016/j.apergo.2020.103121, 32501250

[44] Jakobsen MD Vinstrup J Andersen LL. Factors associated with high physical exertion during healthcare work: cross-sectional study among healthcare workers. Work. (2022) 71:881–8. doi: 10.3233/WOR-213647, 35275592

[45] Zamani Z Joy T Worley J. Optimizing nurse workflow efficiency: an examination of nurse walking behavior and space accessibility in medical surgical units. HERD. (2024) 17:269–89. doi: 10.1177/19375867241237509, 38563318

[46] Chidambaram V Gopalsamy MM Kanchan BK Mouleeswaran S. A holistic methodology for mitigating awkward postural risks: evidence from south Indian small-scale industries. Work. (2024) 77:1031–45. doi: 10.3233/WOR-230210, 37781854

[47] Hashemi F Eghbali SR Mallory-Hill S Hamedi M. A method for prioritizing the modification of ergonomic and physical aspects of the workplace to enhance overall worker satisfaction in control Centre buildings. Int J Occup Saf Ergon. (2021) 27:323–35. doi: 10.1080/10803548.2021.1872334, 33843475

[48] Ibrahim El-Sayed AA Ramadan Asal MG Farghaly Abdelaliem SM Alsenany SA Elsayed BK. The moderating role of just culture between nursing practice environment and oncology nurses’ silent behaviors toward patient safety: a multicentered study. Eur J Oncol Nurs. (2024) 69:102516. doi: 10.1016/j.ejon.2024.102516, 38402719

[49] Mokarami H Eskandari S Cousins R Salesi M Kazemi R Razeghi M . Development and validation of a nurse station ergonomics assessment (NSEA) tool. BMC Nurs. (2021) 20:83. doi: 10.1186/s12912-021-00600-8, 34059027 PMC8165804

[50] Bevan V Blake P Radwan RN Azzopardi E. Sharps and needlestick injuries within the operating room: risk prone procedures and prevalence meta-analysis. J Perioper Pract. (2023) 33:200–10. doi: 10.1177/17504589221103810, 36597950

[51] De Carli G Agresta A Lecce MG Marchegiano P Micheloni G Sossai D . Prevention from sharp injuries in the hospital sector: an Italian National Observatory on the implementation of the council directive 2010/32/EU before and during the COVID-19 pandemic. Int J Environ Res Public Health. (2022) 19:11144. doi: 10.3390/ijerph191711144, 36078860 PMC9518081

[52] Thompson BM Cook CB. Unsafe sharps disposal among insulin-using patients with diabetes mellitus: an emerging global crisis. J Diabetes Sci Technol. (2022) 16:1376–80. doi: 10.1177/19322968211059851, 34852676 PMC9631533

[53] Kaur M Mohr S Andersen G Kuhnigk O. Needlestick and sharps injuries at a German university hospital: epidemiology, causes and preventive potential - a descriptive analysis. Int J Occup Med Environ Health. (2022) 35:497–507. doi: 10.13075/ijomeh.1896.01854, 35661161 PMC10464819

[54] Sarı H Dayan S Balkan H Çiçek Y Özel M. Assessment of sharps penetrating injury, mucosal exposure and compliance with standard precautions of health workers at a University Hospital in Turkey. Saudi Med J. (2023) 44:588–93. doi: 10.15537/smj.2023.44.6.20220898, 37343996 PMC10284234

[55] Alhomayani KM Aljohani GS Alzahrani AN Alwagdani SA Bukhary HA Aljuaid FI. Prevalence, risk factors, and prevention strategies for intraoperative sharp object injuries among orthopedic surgeons: a cross-sectional study. Medicine (Baltimore). (2025) 104:e44975. doi: 10.1097/MD.0000000000044975, 41088712 PMC12517843

[56] Abdelbagi AY Widaa Mohamed AA Yousif ASA Ahmed Osman Mohamed A Abdalla M Saeed Abdelrahim Saeed S . Awareness and perspectives of doctors on needle stick injuries at Port Sudan teaching hospital: a quality improvement project. Cureus. (2025) 17:e95617. doi: 10.7759/cureus.95617, 41322947 PMC12659922

[57] Wong A Nguyen H Eley R Sinnott M. Purchase data: a proxy for safety status. J Hosp Infect. (2020) 105:657–8. doi: 10.1016/j.jhin.2020.05.004, 32389708

[58] Karahan A Avcı Işık S Çevik B Budak Ertürk E Çevik Aydın F Böke Kılıçlı A . Determination of thermal comfort among nurses working with personal protective equipment in COVID-19 clinics. Int J Nurs Pract. (2022) 28:e13112. doi: 10.1111/ijn.13112, 36289017 PMC9874853

[59] Yánez Benítez C Güemes A Aranda J Ribeiro M Ottolino P Di Saverio S . Impact of personal protective equipment on surgical performance during the COVID-19 pandemic. World J Surg. (2020) 44:2842–7. doi: 10.1007/s00268-020-05648-2, 32564140 PMC7305697

[60] Kurtz CE Peng Y Jesso M Sanghavi H Kuehl DR Parker SH. Using a human factors-centric approach to development and testing of a face shield designed for health care workers: a COVID-19 case study for process and outcomes. Am J Infect Control. (2022) 50:306–11. doi: 10.1016/j.ajic.2021.10.033, 34774896 PMC8861890

[61] Shoaib M Jamshaid H Mishra RK Iqbal K Müller M Chandan V . Flammability and thermoregulation performance of multilayer protective clothing incorporated with phase change materials. Materials (Basel). (2024) 17:5826. doi: 10.3390/ma17235826, 39685264 PMC11641941

[62] Xu R Yang L Qin Z. Design, manufacture, and testing of customized sterilizable respirator. J Mech Behav Biomed Mater. (2022) 131:105248. doi: 10.1016/j.jmbbm.2022.105248, 35525065 PMC9577475

[63] Lou MF. Safe working environments: the foundation of patient safety. Hu Li Za Zhi. (2022) 69:4–6. doi: 10.6224/JN.202210_69(5).01, 36127752

[64] Gong J Chen Y Liu D Cai H Jiang L Wang H. Multifunctional ag-PDA@PCM composite eutectogel based electronic skin for personal thermal management and a machine learning assisted wearable sensor. J Mater Chem B. (2025) 13:14047–62. doi: 10.1039/d5tb01479d, 41065068

[65] Rahman A Duan Y Symonds-Brown H Salma J Estabrooks CA. Care aides compassion fatigue, burnout, and compassion satisfaction related to long-term care (LTC) working environment. J Appl Gerontol. (2026) 45:3–16. doi: 10.1177/07334648251328400, 40126450 PMC12681364

[66] Poncette AS Wunderlich MM Spies C Heeren P Vorderwülbecke G Salgado E . Patient monitoring alarms in an intensive care unit: observational study with do-it-yourself instructions. J Med Internet Res. (2021) 23:e26494. doi: 10.2196/26494, 34047701 PMC8196351

[67] Fernandes C Miles S Lucena CJP. Detecting false alarms by analyzing alarm-context information: algorithm development and validation. JMIR Med Inform. (2020) 8:e15407. doi: 10.2196/15407, 32432551 PMC7270842

[68] Cecconi M Hutanu AL Beard J Gonzalez-Pizarro P Ostermann M Batchelor A . Unlocking opportunities to transform patient care: an expert insight on limitations and opportunities in patient monitoring. Intensive Care Med Exp. (2025) 13:24. doi: 10.1186/s40635-025-00733-z, 39984790 PMC11845334

[69] Nyarko BA Giuliano KK. Beyond monitors: intravenous smart pump alarm fatigue as a safety concern. AACN Adv Crit Care. (2025) 36:252–7. doi: 10.4037/aacnacc2025136, 40907102

[70] Walzer S Schön I Pfeil J Klemm S Ziegler S Schmoor C . Nurses' perspectives and experiences of using a bed-exit information system in an acute hospital setting: mixed methods study. JMIR Form Res. (2025) 9:e64444. doi: 10.2196/64444, 39908092 PMC11840387

[71] Khanna AK Flick M Saugel B. Continuous vital sign monitoring of patients recovering from surgery on general wards: a narrative review. Br J Anaesth. (2025) 134:501–9. doi: 10.1016/j.bja.2024.10.045, 39779421

[72] Kim S Cho S Park Y. Daily microbreaks in a self-regulatory resources lens: perceived health climate as a contextual moderator via microbreak autonomy. J Appl Psychol. (2022) 107:60–77. doi: 10.1037/apl0000891, 33646798

[73] Tsunemi M Matsuzaki I Hori Y Hayashi K Tamada H Yamada S . The one-minute triple stretch reduces musculoskeletal discomfort in endoscopic assistants: a crossover trial with motion analysis. Dig Endosc. (2026) 38:e70040. doi: 10.1111/den.70040, 41077957 PMC12745916

[74] Gleave A Shah A Giff A Perera GC Sommer DD Mountjoy M . Promoting longevity in surgical careers: a narrative review and fitness program to reduce occupational pain. J Surg Educ. (2025) 82:103512. doi: 10.1016/j.jsurg.2025.103512, 40393346

[75] Laulan P Fernandez MLG Abet E Dimet J Rimmele U. The flush model: a novel framework to manage surgeons’ mental fatigue and cognitive load. Ann Surg Open. (2025) 6:e581. doi: 10.1097/AS9.0000000000000581, 40557351 PMC12185093

[76] Fox S Dall’Ora C Young M. Fatigue risk management in healthcare: a scoping literature review. Int J Nurs Stud. (2025) 174:105282. doi: 10.1016/j.ijnurstu.2025.105282 41273891

[77] Labrague LJ. Compassion fatigue mediates the relationship between workplace safety climate, career satisfaction, and turnover intention among nurses: a cross-sectional study. Worldviews Evid-Based Nurs. (2025) 22:e70073. doi: 10.1111/wvn.70073, 40958378

[78] Brogaard L Hvidman L Esberg G Finer N Hjorth-Hansen KR Manser T . Teamwork and adherence to guideline on newborn resuscitation-video review of neonatal interdisciplinary teams. Front Pediatr. (2022) 10:828297. doi: 10.3389/fped.2022.828297, 35265565 PMC8900704

[79] Chappell D Neuhaus C Kranke P. Optimal care for mother and child: safety in obstetric anaesthesia. Best Pract Res Clin Anaesthesiol. (2021) 35:41–51. doi: 10.1016/j.bpa.2020.04.001, 33742577

[80] Hildreth AF Cole R Henderson J Shen C. Time is a tool: evaluation of a prolonged casualty care curriculum with a focus on temporal fidelity. Mil Med. (2025) 190:e1727–33. doi: 10.1093/milmed/usaf017, 39836376

[81] Edwards ST Johnson A Park B Eiff P Guzman CEV Gordon L . "what we're doing now…is more than water cooler": perspectives of primary care leaders on leading through (and beyond) COVID-19. J Gen Intern Med. (2024) 39:239–46. doi: 10.1007/s11606-023-08373-3, 37582949 PMC10853095

[82] Cho H Sagherian K Scott LD Steege LM. Occupational fatigue, workload and nursing teamwork in hospital nurses. J Adv Nurs. (2022) 78:2313–26. doi: 10.1111/jan.15246, 35396873

[83] Nemati-Vakilabad R Ebadi E Homaei A Hoseini S Mirzaei A. The relationship between perceived nursing workload and occupational fatigue in clinical nurses: the moderating role of nursing teamwork. J Clin Nurs. (2025) 34:4132–41. doi: 10.1111/jocn.17616, 39654037

[84] Germain A Wolfson M Pulantara IW Wallace ML Nugent K Mesias G . Prototyping apps for the management of sleep, fatigue, and behavioral health in austere far-forward environments: development study. J Med Internet Res. (2023) 25:e40640. doi: 10.2196/40640, 37639304 PMC10495854

[85] Etheridge JC Moyal-Smith R Lim SR Yong TT Tan HK Sonnay Y . Utility of a device briefing tool to improve surgical safety. J Surg Res. (2022) 280:218–25. doi: 10.1016/j.jss.2022.07.018, 36007480

[86] Malet D Falzon P Vidal-Gomel C. Training in “reasoned handling care”: (re)discovering meaning in work. Rech Soins Infirm. (2022) 148:66–78. doi: 10.3917/rsi.148.0066, 36102078

[87] van den Kroonenberg DL Went J Jager A Garrido-Utrilla A Trappenburg JCA Postema AW . Developing a training for 3D transrectal multiparametric ultrasound of the prostate: a human factors engineering approach. Expert Rev Med Devices. (2025) 22:361–7. doi: 10.1080/17434440.2025.2473632, 40040313

[88] Dondin M Baeza-Velasco C. Joint hypermobility and fatigue are associated with injuries in a group of preprofessional ballet dancers. J Dance Med Sci. (2023) 27:80–6. doi: 10.1177/1089313X231177173, 37300373

[89] Liu H Huang H Li M Mao P Zhang A Sun Y . The effect of "online-simulation-bedside" three-step teaching method in team cardiopulmonary resuscitation skills training of emergency department and critical care nursing interns-an analysis based on Kirkpatrick model. J Nurs Manag. (2025) 2025:8624274. doi: 10.1155/jonm/8624274, 40223890 PMC11985234

[90] Cao Z Zhu J Wang Z. Analysis of HFE impact of COVID-19 on OHS in construction enterprises. Heliyon. (2025) 11:e41275. doi: 10.1016/j.heliyon.2024.e41275, 39811363 PMC11730245

[91] Torossian MR Chung J Mamo SK Jacelon CS. Examining a fatigue management model in older individuals. Rehabil Nurs. (2022) 47:50–9. doi: 10.1097/RNJ.0000000000000360, 35234405

[92] Finn HT Kennedy DS Green S Taylor JL. Fatigue-related feedback from calf muscles impairs knee extensor voluntary activation. Med Sci Sports Exerc. (2020) 52:2136–44. doi: 10.1249/MSS.0000000000002362, 32936591

[93] Takizawa M Nakayama N Ohishi Y Tanaka K Noguchi R Saito Y . Physicians' perspectives on prescription alerts: a journey towards reducing fatigue. Cureus. (2025) 17:e86996. doi: 10.7759/cureus.86996, 40741590 PMC12307096

[94] Pula G Moretti P Ritacco I Caramanico G Filippis G Magnone R . Occupational stress and its association with affective disorders in healthcare workers: a cross-sectional study. Psychiatr Danub. (2025) 37:385–90. 40982856

[95] Dalmasso G Di Prinzio RR Gilardi F De Falco F Vinci MR Camisa V . Effectiveness of psychological support to healthcare workers by the occupational health service: a pilot experience. Healthcare. (2021) 9:732. doi: 10.3390/healthcare9060732, 34198556 PMC8231947

[96] Wang B Bai Y Wu S Lin W Guo J. Association between occupational burnout and psychological symptoms among Chinese medical staff: moderating role of social support. Psychol Health Med. (2024) 29:1265–80. doi: 10.1080/13548506.2023.2299666, 38166576

[97] Yao G Mu B Huang Z Feng Y Li S Wang J. Sleep quality and mental health after bloodborne occupational exposure among medical staff in a new crown square cabin: chain mediation of social support and psychological resilience. Wei Sheng Yan Jiu. (2025) 54:631–7. doi: 10.19813/j.cnki.weishengyanjiu.2025.04.015 40695764

[98] Bouleh PG Allen SJ Hammer LB. Family-supportive supervisor behaviors and psychological distress: a secondary analysis across four occupational populations. Int J Environ Res Public Health. (2022) 19:7845. doi: 10.3390/ijerph19137845, 35805503 PMC9265756

[99] Sagherian K Steege LM Cobb SJ Cho H. Insomnia, fatigue and psychosocial well-being during COVID-19 pandemic: a cross-sectional survey of hospital nursing staff in the United States. J Clin Nurs. (2023) 32:5382–95. doi: 10.1111/jocn.15566, 33219569 PMC7753687

[100] Saleh MO Eshah NF Rayan AH. Empowerment predicting nurses’ work motivation and occupational mental health. SAGE Open Nurs. (2022) 8:23779608221076811. doi: 10.1177/23779608221076811, 35224187 PMC8874176

[101] Xie D Zhu X Zhang X Jiang Z Liu T. The impact of support from emergency nurse organizations on compassion fatigue: the mediating role of psychological capital. Front Public Health. (2025) 13:1551381. doi: 10.3389/fpubh.2025.1551381, 40352829 PMC12061964

[102] Marinho PS Alves LVV Carvalho TGB Faria MGA. Protective strategies against occupational stress among health professionals during the COVID-19 pandemic. Rev Bras Med Trab. (2024) 22:e20221016. doi: 10.47626/1679-4435-2022-1016, 39371281 PMC11452111

[103] Zhang N Zhou W Xiao A Weng S Zhang L Zhu D . Psychological interventions mitigated occupational stress in high-risk workers in Shenzhen, China. Front Public Health. (2025) 13:1636004. doi: 10.3389/fpubh.2025.1636004, 40756408 PMC12313477

[104] McGlynn C Choudhury A. Safety and mental health challenges in emergency medical services: a qualitative investigation of rural paramedic experiences. IISE Trans Occup Ergon Hum Factors. (2025) 13:1–13. doi: 10.1080/24725838.2025.2600979, 41451684

[105] Monesi A Imbriaco G Mazzoli CA Giugni A Ferrari P. In-situ simulation for intensive care nurses during the COVID-19 pandemic in Italy: advantages and challenges. Clin Simul Nurs. (2022) 62:52–6. doi: 10.1016/j.ecns.2021.10.005, 34721739 PMC8542439

[106] Kelly LA Schaeffer R Roe S Buchda VL. Using text messages to support nurse well-being. Nurs Adm Q. (2021) 45:338–45. doi: 10.1097/NAQ.0000000000000490 34469392

[107] Kim J Won J Lee Y. Use of a generative pre-trained transformer-based virtual patient for health assessment and communication training in nursing education: a mixed-methods study. Nurse Educ Pract. (2025) 88:104536. doi: 10.1016/j.nepr.2025.104536, 40902283

[108] Delfgaauw J Gameren MV Voorn PB Bossen D Olij BF Visser B . The cost-effectiveness of the Dutch in balance fall prevention intervention compared to exercise recommendations among community-dwelling older adults with an increased risk of falls: a randomized controlled trial. PLoS One. (2025) 20:e0339497. doi: 10.1371/journal.pone.0339497, 41468365 PMC12752955

[109] Yuan X Wang P Liu J. A novel intelligent approach for infection protection using a multidisciplinary collaboration in regional general hospitals. Sci Rep. (2025) 15:22639. doi: 10.1038/s41598-025-06329-7, 40594409 PMC12218119

[110] Bozzani FM Sumner T Mudzengi D Gomez GB White R Vassall A. Informing balanced investment in services and health systems: a case study of priority setting for tuberculosis interventions in South Africa. Value Health. (2020) 23:1462–9. doi: 10.1016/j.jval.2020.05.021, 33127017 PMC7640941

[111] Olinski C Norton CE. Implementation of a safe patient handling program in a multihospital health system from inception to sustainability: successes over 8 years and ongoing challenges. Workplace Health Saf. (2017) 65:546–59. doi: 10.1177/2165079917704670, 28703044

[112] Colpaert K Claus B Somers A Vandewoude K Robays H Decruyenaere J. Impact of computerized physician order entry on medication prescription errors in the intensive care unit: a controlled cross-sectional trial. Crit Care. (2006) 10:R21. doi: 10.1186/cc3983, 16469126 PMC1550814

[113] Abdullah Sharin I Jinah N Bakit P Adnan IK Zakaria NH Mohmad S . Psychoeducational burnout intervention for nurses: protocol for a systematic review. JMIR research protocols. (2024) 13:e58692. doi: 10.2196/58692, 39348680 PMC11474121

[114] Sedile R Zizza A Bastiani L Carluccio E Marrazzi M Bellandi T . Understanding the second victim phenomenon among healthcare Workers in an Italian Hospital. Eur J Investig Health Psychol Educ. (2024) 14:3073–86. doi: 10.3390/ejihpe14120201, 39727509 PMC11675350

[115] Sarioğlu E Amarat M. The relationship between alarm fatigue and medical error tendency in intensive care unit nurses: the mediating affect of role overload. Nurs Crit Care. (2025) 30:e70121. doi: 10.1111/nicc.70121, 40704558 PMC12288113

[116] Koomen E Webster CS Konrad D van der Hoeven JG Best T Kesecioglu J . Reducing medical device alarms by an order of magnitude: a human factors approach. Anaesth Intensive Care. (2021) 49:52–61. doi: 10.1177/0310057X20968840, 33530699 PMC7905747

[117] Ruppel H Pohl E Rodriguez-Paras C Froh E Perry K McNamara M . Clinician perspectives on specifications for metrics to inform pediatric alarm management. Biomed Instrum Technol. (2023) 57:18–25. doi: 10.2345/0899-8205-57.1.18, 37084247 PMC10512991

[118] Alotaibi JS. Causes of medication administration errors and barriers to reporting as perceived by nurses in Saudi Arabia: a qualitative study. Belitung Nurs J. (2024) 10:215–21. doi: 10.33546/bnj.3249, 38690308 PMC11056835

[119] Mahmoud HA Thavorn K Mulpuru S McIsaac D Abdelrazek MA Mahmoud AA . Barriers and facilitators to improving patient safety learning systems: a systematic review of qualitative studies and meta-synthesis. BMJ Open Qual. (2023) 12:e002134. doi: 10.1136/bmjoq-2022-002134, 37012003 PMC10083845

[120] Mishali M Sheffer N Negev M. Challenges and dynamics in reporting medical device incidents: a qualitative study. Front Health Serv. (2025) 5:1720494. doi: 10.3389/frhs.2025.1720494, 41450481 PMC12727643

[121] Ghahramani A Samadi Z Mansouri M Aghaei F. Investigating the culture and practice of reporting occupational incidents in Iranian industries: a mixed-methods study. BMC Public Health. (2025) 25:4364. doi: 10.1186/s12889-025-25332-1, 41469964 PMC12754953

[122] Yan M Chen W Wang J Zhang M Zhao L. Characteristics and causes of particularly major road traffic accidents involving commercial vehicles in China. Int J Environ Res Public Health. (2021) 18:3878. doi: 10.3390/ijerph18083878, 33917131 PMC8067832

[123] Wang J Fu G Yan M. Analysis of a catastrophic commercial coach crash based on an improved accident causation model: cause classification and lessons learned. Int J Occup Saf Ergon. (2022) 28:659–71. doi: 10.1080/10803548.2020.1759314, 32321377

[124] Uçak A Cebeci F Tat Çatal A. Nurses’ alarm fatigue levels in adult intensive care units and their strategies to reduce fatigue: a convergent parallel design. J Clin Nurs. (2025) 34:1691–703. doi: 10.1111/jocn.17644, 39831580 PMC12037928

[125] Hamzaa HG Abdelaal MH Hussein Ramadan Atta M Abdelnaby HSM Othman AA. Analysis of compassion fatigue among psychiatric nurses and its effect on spiritual and competent care. J Psychiatr Ment Health Nurs. (2025) 32:986–97. doi: 10.1111/jpm.13174, 40231844

[126] Burkev FG Taşci S. Aromatherapy experiences of intensive care nurses regarding sleep and fatigue: a phenomenological study. Nurs Crit Care. (2026) 31:e70305. doi: 10.1111/nicc.70305, 41416440

[127] Bani Hani S Abu Aqoulah EA. Relationship between alarm fatigue and stress among acute care nurses: a cross-sectional study. SAGE Open Nurs. (2024) 10:23779608241292584. doi: 10.1177/23779608241292584, 39493251 PMC11528618

[128] Schumacher AE Zheng P Barber RM Bhoomadevi A Aalipour MA Aalruz H . Global age-sex-specific all-cause mortality and life expectancy estimates for 204 countries and territories and 660 subnational locations, 1950-2023: a demographic analysis for the global burden of disease study 2023. Lancet. (2025) 406:1731–810. doi: 10.1016/S0140-6736(25)01330-3 41092927 PMC12535839

[129] Gmayinaam VU Nortey AN Sedode S Apedo SK Kye-Duodu G Kwabla P . Work-related stress among nurses: a comparative cross-sectional study of two government hospitals in Ghana. BMC Public Health. (2024) 24:2257. doi: 10.1186/s12889-024-19757-3, 39164666 PMC11334492

[130] Descatha A Sembajwe G Pega F Ujita Y Baer M Boccuni F . The effect of exposure to long working hours on stroke: a systematic review and meta-analysis from the WHO/ILO joint estimates of the work-related burden of disease and injury. Environ Int. (2020) 142:105746. doi: 10.1016/j.envint.2020.105746, 32505015

[131] Li J Pega F Ujita Y Brisson C Clays E Descatha A . The effect of exposure to long working hours on ischaemic heart disease: a systematic review and meta-analysis from the WHO/ILO joint estimates of the work-related burden of disease and injury. Environ Int. (2020) 142:105739. doi: 10.1016/j.envint.2020.105739, 32505014 PMC7339147

[132] Ross A Geiger-Brown J Yang L Flynn S Cox R Wehrlen L . Acute and chronic fatigue in nurses providing direct patient care and in non-direct care roles: a cross-sectional analysis. Nurs Health Sci. (2021) 23:628–38. doi: 10.1111/nhs.12862, 34145719 PMC8543448

[133] Adams J Cotton J Brumby S. Agricultural health and medicine education-engaging rural professionals to make a difference to farmers’ lives. Aust J Rural Health. (2020) 28:366–75. doi: 10.1111/ajr.12637, 32596870

[134] Fazzini B McGinley A Stewart C. A multidisciplinary safety briefing for acutely ill and deteriorating patients: a quality improvement project. Intensive Crit Care Nurs. (2023) 74:103331. doi: 10.1016/j.iccn.2022.103331, 36208975

[135] Glarcher M Rihari-Thomas J Duffield C Tuqiri K Hackett K Ferguson C. Advanced practice nurses’ experiences of patient safety: a focus group study. Contemp Nurse. (2025) 61:242–56. doi: 10.1080/10376178.2024.2363911, 38861587

[136] Vinar AL Cipher DJ Ormand M Carlisle B Behan D. Multidisciplinary teamwork perceptions when mobilizing ventilated neurosurgery patients. J Neurosci Nurs. (2023) 55:199–204. doi: 10.1097/JNN.0000000000000726, 37612259

[137] Knight J Richelieu J Velasco JM Lubinsky G Day E Weiss T . Improving the team response to surgical airway emergencies: a simulation-based, multidisciplinary approach to quality improvement. J Surg Educ. (2025) 82:103544. doi: 10.1016/j.jsurg.2025.103544, 40378640

[138] Lim S D’Souza C. A narrative review on contemporary and emerging uses of inertial sensing in occupational ergonomics. Int J Ind Ergon. (2020) 76:102937 33762793 PMC7985982

[139] Asan O Choudhury A. Research trends in artificial intelligence applications in human factors health care: mapping review. JMIR Hum Factors. (2021) 8:e28236. doi: 10.2196/28236, 34142968 PMC8277302

[140] Kelly D Purssell E Wigglesworth N Gould DJ. Electronic hand hygiene monitoring systems can be well-tolerated by health workers: findings of a qualitative study. J Infect Prev. (2021) 22:246–51. doi: 10.1177/17571774211012781, 34880946 PMC8647641

[141] Gurubhagavatula I Barger LK Barnes CM Basner M Boivin DB Dawson D . Guiding principles for determining work shift duration and addressing the effects of work shift duration on performance, safety, and health: guidance from the American Academy of sleep medicine and the Sleep Research Society. J Clin Sleep Med. (2021) 17:2283–306. doi: 10.5664/jcsm.9512, 34666885 PMC8636361

[142] Giunti G Yrttiaho T Guardado-Medina S Sachinopoulou A Mylonopoulou V Fält J . Feasibility and usability evaluation of a gamified fatigue management mobile application for persons with multiple sclerosis in everyday life. Mult Scler Relat Disord. (2025) 97:106379. doi: 10.1016/j.msard.2025.106379, 40073696

[143] Giunti G Rivera-Romero O Kool J Bansi J Sevillano JL Granja-Dominguez A . Evaluation of more stamina, a mobile app for fatigue management in persons with multiple sclerosis: protocol for a feasibility, acceptability, and usability study. JMIR Res Protoc. (2020) 9:e18196. doi: 10.2196/18196, 32749995 PMC7435635

[144] Morgan KA Wong AWK Walker K Desai RH Knepper TM Newland PK. A mobile phone text messaging intervention to manage fatigue for people with multiple sclerosis, spinal cord injury, and stroke: development and usability testing. JMIR Form Res. (2022) 6:e40166. doi: 10.2196/40166, 36542466 PMC9813815

[145] Dorronzoro-Zubiete E Castro-Marrero J Ropero J Sevillano-Ramos JL Dolores Hernández M Sanmartin Sentañes R . Personalized management of fatigue in individuals with myalgic encephalomyelitis/chronic fatigue syndrome and long COVID using a smart digital mHealth solution: protocol for a participatory design approach. JMIR Res Protoc. (2024) 13:e50157. doi: 10.2196/50157, 38608263 PMC11053387

[146] Sellers MM Fieber J Myers JS Shea J Kelz RR Dowzicky PM. Patient safety reporting systems and surgical safety culture: one culture to rule them all? J Surg Educ. (2026) 83:103769. doi: 10.1016/j.jsurg.2025.103769, 41317508

[147] Hicks S Stavropoulou C. The effect of health care professional disruptive behavior on patient care: a systematic review. J Patient Saf. (2022) 18:138–43. doi: 10.1097/PTS.0000000000000805, 33395017

[148] Madjdian DS Dankwah Badu V Ilboudo G Lallogo VR Dione M van Asseldonk M . Fast food over safe food? A qualitative evaluation of a food safety training intervention for street vendors applying the COM-B model in Ouagadougou, Burkina Faso. PLoS One. (2024) 19:e0313635. doi: 10.1371/journal.pone.0313635, 39570854 PMC11581311

[149] Pimentel J Cockcroft A Andersson N. Impact of co-designed game learning on cultural safety in Colombian medical education: protocol for a randomized controlled trial. JMIR Res Protoc. (2020) 9:e17297. doi: 10.2196/17297, 32442146 PMC7490681

[150] Pimentel J Cockcroft A Andersson N. Impact of game jam learning about cultural safety in Colombian medical education: a randomised controlled trial. BMC Med Educ. (2021) 21:132. doi: 10.1186/s12909-021-02545-7, 33632194 PMC7905593

[151] Duffy C Menon N Horak D Bass GD Talwar R Lorenzi C . Proactive patient safety: focusing on what goes right in the perioperative environment. J Patient Saf. (2023) 19:281–6. doi: 10.1097/PTS.0000000000001113, 36849540

[152] Lyubykh Z Turner N Hershcovis MS Deng C. A meta-analysis of leadership and workplace safety: examining relative importance, contextual contingencies, and methodological moderators. J Appl Psychol. (2022) 107:2149–75. doi: 10.1037/apl0000557, 35298213

[153] Mazur LM Khasawneh A Fenison C Buchanan S Kratzke IM Adapa K . A novel theory-based virtual reality training to improve patient safety culture in the department of surgery of a large academic medical center: protocol for a mixed methods study. JMIR Res Protoc. (2022) 11:e40445. doi: 10.2196/40445, 36001370 PMC9453584

[154] Maoz Breuer R Waitzberg R Breuer A Cram P Bryndova L Williams GA . Work like a doc: a comparison of regulations on residents' working hours in 14 high-income countries. Health Policy. (2023) 130:104753. doi: 10.1016/j.healthpol.2023.104753, 36827717

[155] Pramanda R Partiwi SG Sudiarno A Hamdani. Investigation into the determinants of occupational safety for achieving zero accidents in multi-storey building construction projects: a Bayesian belief network approach. MethodsX. (2025) 15:103508. doi: 10.1016/j.mex.2025.103508, 40726916 PMC12301787

[156] Saleem Maabreh R Mahran GSK Khamies Mohamed N Gamal Abd-Elhamed A. To nap or not to nap? Medical managers’ views on night shift fatigue management. Crit Care Nurs Q. (2025) 48:373–80. doi: 10.1097/CNQ.0000000000000574, 41021681

[157] Mahran GSK Abu Aqoulah EA Seleem EAES Hawash MAE Ahmed RDM. Wake up call: a qualitative study of nursing and medical managers’ perceptions and support for nurses’ night shift napping in intensive care units. Nurs Crit Care. (2025) 30:e70056. doi: 10.1111/nicc.70056, 40368842

